# Recent Advances in UHMWPE/UHMWPE Nanocomposite/UHMWPE Hybrid Nanocomposite Polymer Coatings for Tribological Applications: A Comprehensive Review

**DOI:** 10.3390/polym13040608

**Published:** 2021-02-18

**Authors:** Mohammed Abdul Samad

**Affiliations:** Department of Mechanical Engineering, King Fahd University of Petroleum and Minerals, Dhahran 31261, Saudi Arabia; samad@kfupm.edu.sa

**Keywords:** UHMWPE coatings, tribology, friction, wear

## Abstract

In the recent past, polymer coatings have gained the attention of many researchers due to their low cost, their ability to be coated easily on different substrates, low friction and good anti-corrosion properties. Various polymers such as polytetrafluroethylene (PTFE), polyether ether ketone (PEEK), polymethylmethacrylate (PMMA), polyurethane (PU), polyamide (PA), epoxy and ultra-high molecular weight polytheylene (UHMWPE) have been used to develop these coatings to modify the surfaces of different components to protect them from wear and corrosion. However, among all these polymers, UHMWPE stands out as a tribologist’s polymer due to its low friction and high wear resistance. These coatings have found their way into applications ranging from microelectro mechanical systems (MEMS) to demanding tribological applications such as bearings and biomedical applications. Despite its excellent tribological properties, UHMWPE suffers from limitations such as low load bearing capacity and low thermal stability. To overcome these challenges researchers have developed various routes such as developing UHMWPE composite and hybrid composite coatings with several types of nano/micro fillers, developing composite films system and developing dual film systems. The present paper is an effort to summarize these various routes adopted by different researchers to improve the tribological performance of UHMWPE coatings.

## 1. Introduction

Coating is a surface engineering process in which a material is deposited or applied to the surface of a component to improve its properties. The improvements may be in areas as diverse as visual appearance, optical properties, wettability, corrosion resistance or tribological performance. The two main goals in the use of surface engineering for tribological applications are to increase the wear resistance of the component and alter its frictional behavior. Various hard coatings have been used for the protection of contacting surfaces in mechanical systems, such as diamond-like carbon (DLC) coatings, metal carbide coatings (CrC, TiC, WC), molybdenum disulphide (MoS_2_) coatings and PVD coatings (ZrN, TiAlN, CrAlN, ZrC, W–C:H, WC/C, TiO_2_, Al_2_O_3_) [1,2,3,4,5,6]. Although these coatings are helpful in providing high wear resistance, they still suffer from limitations such as high thermal stress, high friction, incompatibility with lubricants, poor adhesion with the substrates and sensitivity to the environment [7,8]. There is also a concern regarding the generation of the worn debris particles from the hard coatings, which can damage the entire tribological system adversely. CVD coatings often display high residual compressive stresses [9]. The high residual stress level in the coatings brings both advantages and problems. Compressive stresses will reduce the propagation of cracks in the coating by pressing together the two surfaces formed at the crack tip. If there is a residual compressive stress in the coating and the component is subject to tension, the coating is protected from tensile stresses, but if the substrate is not perfectly smooth, a high residual stress in combination with a weak interface can cause the coating to spall off spontaneously even before the coated component has been put into use [3].

The ever-increasing demand for improved wear life and reduced coefficients of friction in order to facilitate energy conservation has motivated many researchers to investigate the role of polymer coatings in various tribological applications. Due to the fact that the PVD or CVD coating techniques are expensive and difficult to handle, polymer coatings with their ability to be coated using simple techniques and their cost effectiveness present a very good alternative protective technology. Among the different available polymers, polytetrafluoroethylene (PTFE) [10,11,12], polyether ether ketone (PEEK) [13,14,15,16], epoxy [17,18,19,20] and polyamide [21,22], have been extensively used for coating systems in their pristine form or in the form of composites. However, from the tribology perspective, all the above mentioned polymers have conflicting characteristics in terms of wear rate and coefficient of friction. PTFE, which is termed as a solid lubricant shows a low coefficient of friction, usually in the range of 0.08 to 0.1, but shows a high wear rate. Even though PEEK is considered as high-performance polymer because of its higher temperature resistance, its coefficient of friction is on the higher side, in the range of 0.18 to 0.25, and it shows higher wear rates [23].

**Figure 1:**
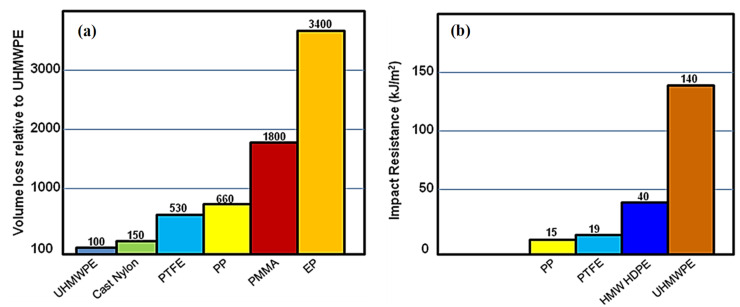
(**a**) Volume loss of most common polymers relative to UHMWPE (**b**) Impact resistance of UHMWPE as compared to other polymers. Adapted from [24].

Ultra-high molecular weight polyethylene (UHMWPE) is one such engineering polymer, which is considered as a tribologist’s polymer because of its excellent tribological properties such as low coefficient of friction (0.08–0.12), high wear/abrasion resistance, high impact resistance, high toughness, high corrosion resistance, high water absorption resistance and biocompatibility. Figure 1 shows the volume loss of other polymers and its impact strength in comparison to other engineering polymers.

Because of the excellent abovementioned properties, UHMWPE has found its way in various demanding applications ranging from microelectromechanical systems (MEMS) to mechanical bearings and biomedical applications both in its bulk form and in the form of coatings.

**Figure 2:**
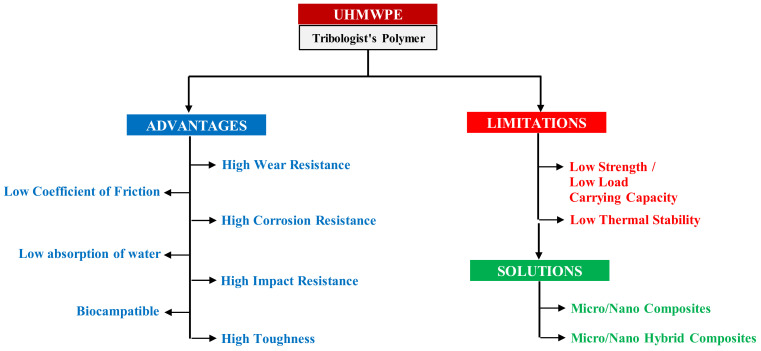
UHMWPE as a Tribo-Material: Advantages, Limitations and Solutions.

UHMWPE is a thermoplastic, semi-crystalline, linear homopolymer which is made up of hydrogen and carbon. Table 1 summarizes the structure and a few important properties of UHMWPE. Despite its excellent mechanical and tribological properties, UHMWPE does suffer from a few limitations, such as low thermal stability and low load bearing capacity. Figure 2 summarizes the advantages, limitations and some possible solutions to overcome the limitations for using UHMWPE as a tribomaterial, either in bulk form or in the form of a tribological protective coating.

Various researchers have conducted extensive studies to improve the mechanical, thermal, tribological and surface properties of UHMWPE. Different routes such as improving cross-linking [26,27] or crystallinity percentage [28,29], through irradiation [30], surface modification through plasma treatment [31,32] or introducing effective textures [33,34], and reinforcements with different fillers [35,36,37,38] have been used for enhancing the properties of bulk UHMWPE. Numerous review articles have already been published highlighting and summarizing the various advancements in the field of improving the mechanical, tribological, and thermal properties of bulk UHMWPE for different applications [39,40,41]. However, in spite of the major advances made in using UHMWPE as protective coatings for demanding tribological and biomedical applications, there is no single review article which has tried to summarize this progress. Hence, this review article aims to fill that gap in the literature, to summarize the various works conducted on UHMWPE coatings and comprehensively present the different coating techniques, substrate pre-treatments, micro/nano fillers used and their different targeted applications. 

**Figure 3:**
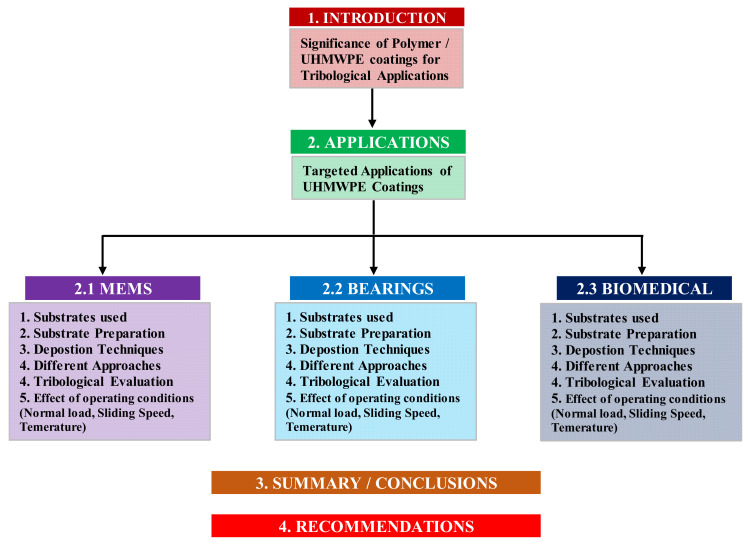
A bird’s view of the arrangement of this article.

It is to be noted that the tribological response of polymers in the bulk form and in the form of coatings differ quite significantly. In the bulk form, polymers may show lower dimensional stability because of high thermal expansion as the local temperature and the frictional contacts in the bulk polymers may be higher due to low dissipation of heat as compared to the thin polymer coatings. Moreover, the tribological response of the coatings depends upon many other factors such as the properties of the substrate and adhesion to the substrate, which in turn depends upon the cleanliness and the pre-treatment procedures. It also depends upon the operating conditions such as the normal load, sliding peed and temperatures. The post-deposition procedures also play an important role in the tribological performance of the coatings. Furthermore, the types of fillers used to fabricate composite coatings also have a significant effect on the overall properties of the coatings. Hence, it is very essential to have an overview of all these important factors, which shall be discussed in detail in the following sections. Figure 3, shows a bird view of the arrangement of this article for the easy understanding of the reader with respect to the UHMWPE coatings.

## 2. UHMWPE Coatings for Different Applications

As mentioned above, UHMWPE coatings have found their way into a various range of tribological applications. However, on conducting an extensive literature survey, the applications for which the UHMWPE coatings have been developed by various researchers can be broadly classified into three categories, such as microelectromechanical systems (MEMS), mechanical bearing systems and biomedical applications. The following sections shall try to discuss the various aspects of the UHMWPE coating technology for each of these applications in detail and present a comprehensive summary.

### 2.1. UHMWPE Coatings for MEMS Applications

Microelectromechanical systems or MEMS are devices with sizes ranging from less than one micron to several millimetres, consisting of miniature mechanical moving parts (0.001 to 0.1 mm) with integrated electronics. MEMS devices include sensors, actuators, motors, pumps and microelectronics. MEMS are usually manufactured using microfabrication technologies and silicon (Si) is one of the backbone materials for these devices [42]. Due to the high surface area to volume ratios in MEMS, because of their miniature sizes, as compared to the larger mechanical devices, surface/adhesive forces play an important role in controlling the tribological performance of the contacting parts. However, bare Si is a very poor tribological material as it shows high friction, high adhesion and high wear [43]. To improve the tribological performance of Si, researchers have taken up many approaches, such as developing molecular lubricants and depositing ultra-thin films/coatings [44,45,46,47,48,49,50,51]. Even though most of these developed coatings show low coefficients of friction, they still suffer from the problem of high wear. 

Another challenge faced by researchers when making UHMWPE coatings suitable for the MEMS applications is how to keep the thickness of the coating as thin as possible without compromising on the wear durability, frictional properties and load bearing capacity. Hence, they have used different approaches to overcome this challenge, which are listed below.

#### 2.1.1. Approach 1: A Bi-Layer Composite Coating of UHMWPE with an Overcoat of Perfluoropolyether (PFPE) Lubricant

Satynarayana et al. [52] made the first attempt of using low friction and high wear resistant UHMWPE coatings for MEMS applications. They used the approach of depositing a composite dual film consisting of UHMWPE as the first layer, overcoated with an ultra-thin layer of perfluoropolyethylene (PFPE) lubricant using a simple dip coating process on a Si wafer. The total thickness achieved for the composite dual film was about 28 µm, which was ensured by controlling the withdrawal speed of the substrate from the coating solution. As mentioned above, one of the problems faced in MEMS is that of stiction, which can be overcome by making the surfaces hydrophobic. Sataynarana et al. were able to obtain a water contact angle (WCA) of 127° (hydrophobic) by depositing the UHMWPE film on the Si substrate as compared to a WCA of 28° (hydrophilic) for a bare Si substrate. Moreover, the WCA increased to 134° after the overcoat of PFPE on the UHMWPE film. Ball-on-disk wear tests conducted on the bare Si substrates and the Si substrates coated with the dual composite film (UHMWPE/PFPE) revealed a significant reduction in coefficient of friction from 0.6 for Si substrate to 0.08 for the substrate coated with the dual film. However, there was a significant increase in the wear life of the Si substrates coated with the dual composite film (UHMWPE/PFPE). The dual film did not fail even until 100,000 cycles as compared to a few cycles for bare Si substrates when tested at a contact pressure of 370 MPa and sliding speeds in the range of 0.04 to 0.08 m/s. They defined the wear life as the number of cycles after which the coefficient of friction exceeded a value of 0.3 or a visible wear scar appeared on the substrate, whichever happened earlier. 

#### 2.1.2. Approach 2: Chemisorption of Polar UHMWPE on Si Substrates with an Intermediate Layer of GPTMS SAM

Satyanarayana et al. [53] presented another approach of chemisorption of UHMWPE coatings on Si substrates for MEMS applications. The objective of this study was to reduce the thickness of the UHMWPE coatings to make them more applicable for MEMS applications without compromising on their friction and the wear life. Hence, in this study, they used polar UHMWPE (molecules of UHMWPE modified with chemical groups such as carboxyl and hydroxyl), which was deposited by a simple dip-coating process on a Si wafer with an glycidoxypropyltrimethoxysilane self-assembled monolayer (GPTMS SAM) intermediate layer. They found that the chemisorption of the polar UHMWPE film on the GPTMS layer significantly improved the WCA, reduced the coefficient of friction and increased the wear life. This was attributed to the enhanced adhesion between Si substrate and the polar UHMWPE coating due to the chemical bonding between the carboxyl and hydroxyl groups of polar UHMWPE molecules and the GPTMS intermediate monolayer, which itself is strongly chemically bonded with the Si substrate by the Si-O-Si bonds. Wear tests were conducted at a load of 0.3 N and a sliding velocity of 0.042 m/s. They also highlighted the importance of a post-heat treatment process of exposing the dual composite film to a temperature of ~110 °C, which enhanced the wear life significantly. They attributed this to the enhanced covalent bonding between the functionalized groups leading to an improved adhesion with the substrate. Table 2 summarizes the important results obtained by Satyanarayan et al. for both the reported approaches.

It can be noted from the above two studies that both the dual composite film approaches resulted in an exceptional wear life coupled with low wettability and low coefficient of friction. All these characteristics are essential for a tribological coating to be successfully employed in MEMS. One of the other key points that has to be noted from the above studies is the effect of adhesion on the wear life of the coating, which is enhanced by the functionalization of the polymer molecules.

#### 2.1.3. Approach 3: A Bi-Layer Composite Coating of UHMWPE with an Intermediate Hard Layer

Further to the above studies, Minn et al. [54] used an approach based on alternative hard/soft composite layers to develop a wear-resistant, multi-layered coating with an excellent load bearing capacity. It is to be noted that the major advantage of soft polymeric coatings is their low shear strength, which also brings along a few limitations such as a large contact area resulting in higher frictional force and low load bearing capacity. However, they proposed that adding a hard layer in between the substrate and the soft polymeric coating would lead to an increase in the load-bearing capacity with the hard layer providing the mechanical support and strength and the overcoat of the soft layer takes care of the low friction. They used diamond-like carbon coating (DLC) as a hard layer (thickness = 50 nm, hardness = 57 GPa, deposited using a filtered cathodic vacuum arc technique) over which a soft coating of UHMWPE was deposited by using a simple dip-coating process. Two sets of wear tests were conducted to evaluate by using a ball-on-disk configuration using a 4 mm diameter silicon nitride ball. They defined the wear life as the number of cycles after which the coefficient of friction exceeded a value of 0.3 or a visible wear scar appeared on the substrate, whichever happened earlier.

In the first set of tests, the thickness of the UHMWPE coating was fixed at 28 µm and the effect of the DLC underlayer on WCA, friction and wear life was evaluated. To further increase the wear life of this bilayer, they used PFPE as an overcoat as well. Table 3 gives a summary of their results. It can be clearly seen from Table 3, that the DLC underlayer had a significant effect on friction and wear life. The Si/UHMWPE sample showed a coefficient of friction (COF) and wear life of 0.18 and ~20,000 cycles as compared to the Si/DLC/UHMWPE sample, which showed a COF and a wear life of 0.13 and ~100,000 cycles. Even with an overcoat of PFPE, the Si/DLC/UHMWPE/PFPE sample did not fail even until 300,000 cycles and showed a significant improvement in the wear life as compared to the sample, which did not have a DLC underlayer (Si/UHMWPE/PFPE). They attributed this improvement to the high load bearing capacity provided by the DLC layer, which is reflected in the better tribological performance.

**Figure 4:**
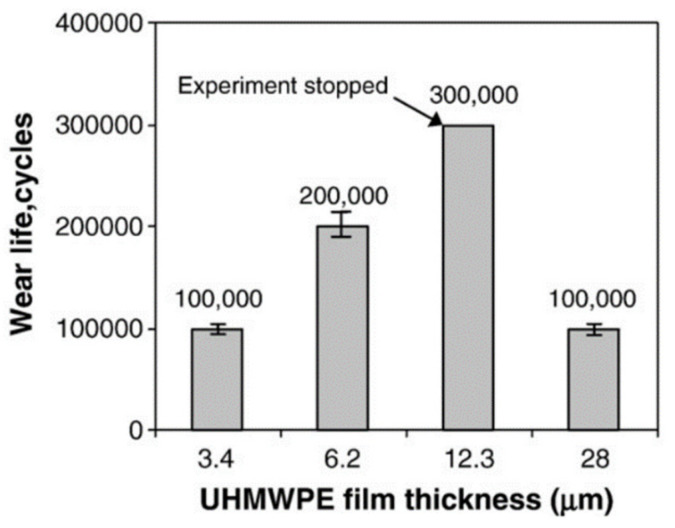
Wear life for different UHMWPE thicknesses for Si/DLC/UHMWPE. Data are averages of three repeated tests. For 12.3 μm thick film there was no failure at 300,000 cycles of sliding when the experiments were stopped due to long test duration [54]. Reproduced with permission from Minn, M. and Sinha, S.K., Surface and Coating Technology; published by Elsevier, 2008.

A second set of wear tests were also conducted on samples with varying UHMWPE coating thicknesses (3.4, 6.2, 12.3 and 28 µm). They found an interesting trend in the wear life of the coatings as a function of their thickness, as shown in Figure 4. They observed that the range of optimum thickness of the UHMWPE coating with a DLC underlayer for the maximum wear life was from 6.2 to 12.3 µm to avoid the large interface/substrate effect on friction and wear (for low film thickness) or, the substantial polymer transfer to the counterface (for high film thickness).

**Figure 5:**
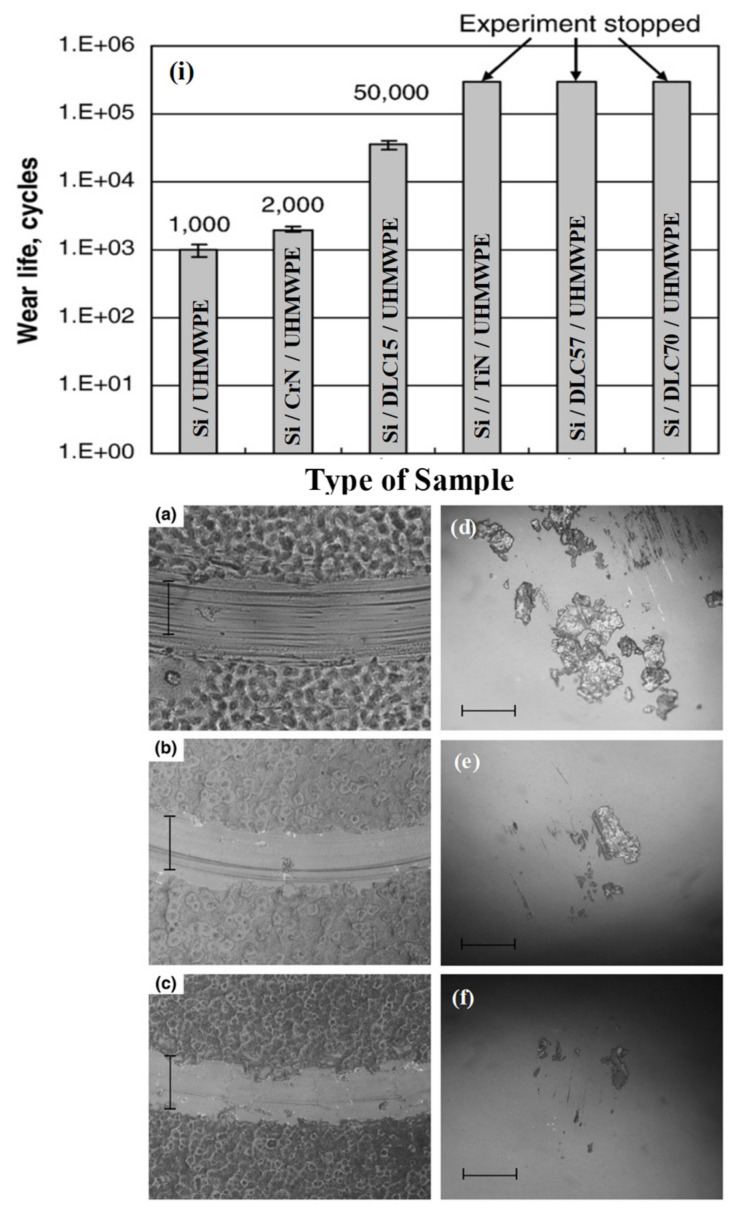
(**i**) Wear life of Si coated with different composite films. [(**a**–**c**)]–Optical images of the wear tracks and [(**d**–**f**)]–Optical images of the counterface Si_3_N_4_ balls after a wear test for 300,000 cycles on UHMWPE coatings deposited on TiN, DLC57 and DLC70 layers, respectively [55]. The applied load was 40 mN and the rotational speed was 500 rpm (linear speed = 0.052 m/s). Reproduced with permission from Myo Minn, Sujeet K Sinha, Thin Solid Films; published by Elsevier, 2010.

Minn et al. [55], further investigated the effect on the tribological performance of the UHMWPE coating deposited on Si substrates with different hard layers such as, chromium nitride (CrN: Hardness = 13.5 GPa), titanium nitride (TiN: hardness = 24 GPa) and diamond like carbon coatings with varying hardness values (DLC: hardness = 15, 57 and 70 GPa). The thickness of the hard layer was kept constant at 50 nm, which was deposited using the filtered cathodic vacuum arc technique. A thin soft layer (thickness = 4 to 5 µm) of UHMWPE was deposited on these hard layers coated Si substrates using a simple dip coating process. As shown in Figure 5, they observed that the wear life of the bilayered coating, increased with the hardness of the intermediate layer. The bilayered coating with TiN (24GPa), DLC (57 GPa) and DLC (70 GPa) showed a wear durability of 300,000 cycles, when the wear tests were conducted at a normal load of 40 mN and a sliding speed of 0.052 m/s. The polymer transfer mechanism, which plays a very important role in determining the tribological performance of polymers was also analyzed as shown in Figure 5. They found out that for the UHMWPE polymer coating with TiN and DLC57 intermediate layers, the transfer was in the form of lumps whereas for DLC70, hardly any transfer was observed. With an increase in the normal load to 70 mN, the bilayered coatings of TiN/UHMWPE, DLC57/UHMWPE and DLC70/UHMWPE showed reduced wear lives of 8000, 22,000 and 120,000 cycles, respectively. However, the bilayered coating with the hardest intermediate layer (DLC70) still showed the highest wear life. Furthermore, an overcoat of a few nanometer thick PFPE layer increased the wear life of all the three above mentioned coatings to one million cycles, when the experiment was stopped.

Minn et al. [56] also investigated the effect of sliding in different directions on a UHMWPE coating deposited on a DLC-modified Si substrate. They showed that the sliding of the ball on the UHMWPE coating induces changes in the crystallinity of the coating which in turn effects the tribological performance. Moreover, the molecular orientation also affects the frictional behavior of the coating. Hence, they concluded that factors such as relative crystallinity and molecular orientation have to be considered while depositing highly lubricious polymer coatings on substrates. 

#### 2.1.4. Approach 4: Interfacial Energy Modifications of the Si Substrate to Improve the Tribological Performance

**Figure 6:**
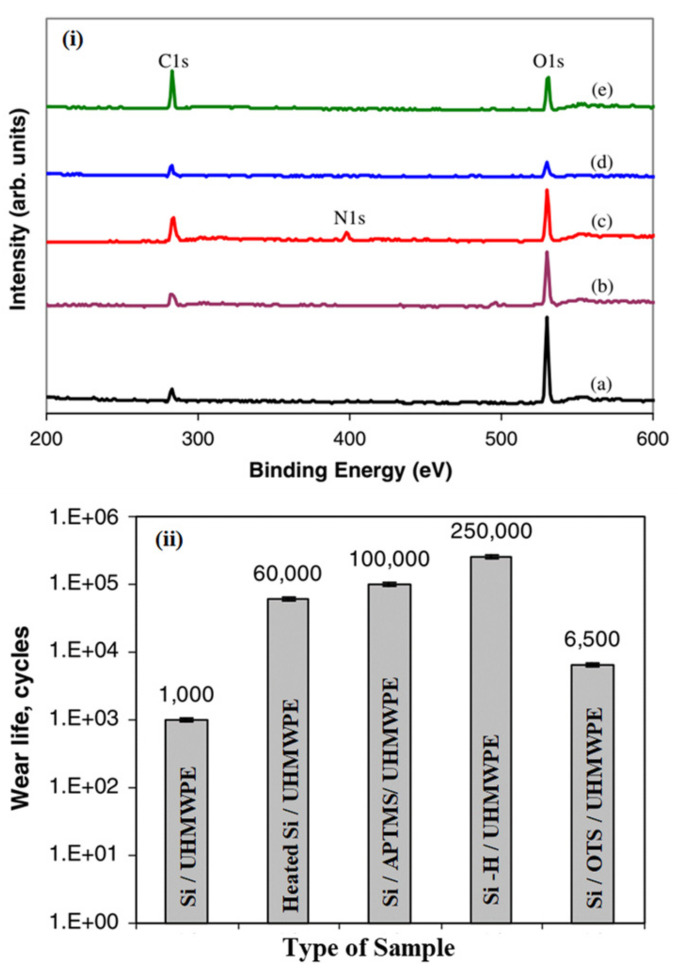
(**i**) XPS spectrum for (**a**) bare Si, (**b**) heated Si, (**c**) Si/APTMS, (**d**) Si-H, (**e**) Si/OTS (**ii**) Wear life behaviour for different samples [57]. Reproduced with permission from Myo Minn, Leong Yonghui Jonathan and Sujeet K Sinha, J. Phys. D: Appl. Phys; published by IOP Publishing, 2008.

Minn et al. [57] also investigated the effect of interfacial energy modifications on the tribological performance of UHMWPE-coated Si. They used five different interfacial layers by physical and chemical methods with different surface energies (hydrophobicity), namely bare Si (i.e., no interfacial modification), heated Si, Si/3-aminopropyltrimethoxysilane (Si/APTMS), hydrogenated Si (Si–H) and Si/octadecyltrichlorosilane (Si/OTS). The water contact angles, which are a measure of the surface energy, were measured for each of these surface-modified Si substrates and were found to be, 21°, 42°, 56°, 71° and 104°, respectively. A 6 µm thick UHMWPE coating was deposited on these surface-modified Si substrates and the tribological performance was evaluated using a ball-on-disk configuration against a 4 mm diameter ball at a normal load of 40 mN and a sliding speed of 0.1 m/s. They observed that the surface with the highest surface energy (bare Si: 21°) and the surface with the lowest surface energy (Si/OTS: 104°) showed low wear lives and high friction coefficients. The hydrogenated Si surface (Si-H: 71°) showed the highest wear life of about 250,000 cycles with low coefficient of friction. Figure 6 shows the wear life of the UHMWPE coating deposited on the various surface modified Si substrates and x-ray photoelectron scope (XPS) spectrum of the different surface modified Si substrates. As can be observed from the XPS spectrum, the bare Si has a large oxygen peak, indicating the presence of oxide and moisture. However, as the surface is modified using different techniques the oxygen peak reduces in intensity and the lowest is shown by hydrogenated Si (Si-H) surface because of the presence of hydrogen termination on the Si surface, which prevents the formation of oxide. They concluded that the wear performance of the UHMWPE coating increases with a decreasing surface energy (increasing water contact angle) and reaches an optimum value after which it starts deteriorating. They attributed this trend to the fact that as the surface energy decreases further after the optimum value, the deposition of the UHMWPE coating also becomes difficult resulting in a non-uniform coating with lower adhesion strength leading to a weak interface.

Encouraged by the above results, which showed a strong dependency of the tribological performance of the UHMWPE coatings on the surface energy of the Si substrates, Minn et al. [57], conducted further experiments by changing the surface energy of the Si substrates by different means. They used air-plasma treatment and a coating of PFPE (3 to 4 nm) to alter the surface energy of the Si substrates before depositing the UHMWPE coating. Based upon the tests, they modeled the shear stress as a function of contact pressure and the pull-off force. They observed that the initial coefficient of friction increased exponentially with an increase in the pull-off force and it decreased with normal load at a constant pull-off force. They concluded that the model would be helpful in predicting the initial coefficient of frictions and help in controlling the same by controlling the surface energy.

#### 2.1.5. Summary

**Figure 7:**
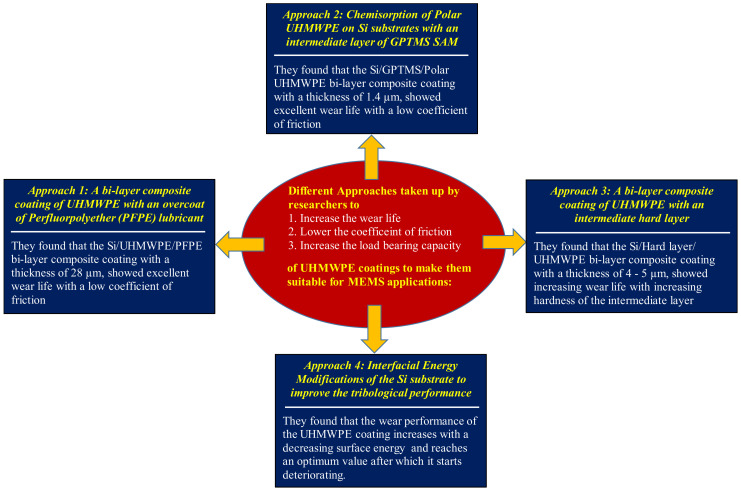
A summary of the different approaches taken up by the researchers to make UHMWPE coatings suitable for MEMS applications.

Figure 7 shows the summary of all the approaches taken up by different research groups to improve the tribological performance of the UHMWPE coatings so as to make them suitable for usage in MEMS applications.

To summarize the literature presented on the application of UHMWPE coatings for MEMS applications, it is to be noted that, a few factors as listed below are very important to obtain sustainable coatings with high durability, low friction and high load bearing capacity. They are:

- *Pre-Treatment of the Si Substrate (Piranha Treatment)*: A proper pre-treatment of the Si substrate so that the surface is devoid of any hydrocarbons or impurities and also increasing the wettability of the surface so that it will help in improving the adhesion of the coating to the substrate, which in turn affects its tribologial performance. In the literature presented above [52,53,54,55,56,57], they used a piranha treatment, which was very effective in cleaning and improving the wettability of the surface. The steps in piranha treatment include the following:
  ○ Si substrates were rinsed for 1 min and ultrasonically cleaned for 15 min each, first with soapy water, second with distilled water and finally with acetone.
  ○ The cleaned substrates were blow-dried with pure nitrogen gas and immersed into a piranha solution (70 vol% H_2_SO_4_ and 30 vol% H_2_O_2_*)* at a temperature of 120 °C for an hour to remove any contaminants.
  ○ After that, the substrates were rinsed again with distilled water and acetone for 1 min each.

- *UHMWPE Coating Procedure (Dip-Coating Process)*: In the literature presented above, all the UHMWPE coatings were deposited using a simple dip-coating process, which helped obtaining coatings with uniform thickness. The thickness of the coating could be controlled by changing the wt.% of UHMWPE powder used to prepare the dip-coating solution by following the procedure listed below:
  ○ The first step in the preparation of the UHMWPE coatings was the dissolution of the right amount (wt.%) of UHMWPE polymer powder into decalin. This is carried out at a higher temperature of 170 °C to 180 °C, which results in complete and uniform dissolution of the polymer.
  ○ Magnetic stirring is used during the heating process in order to enhance the rate of dissolution.
  ○ After ensuring the complete dissolution of the polymer powder, dip-coating is carried out almost immediately using a dip-coating machine.
  ○ The dip-coating is carried out at a constant dipping and withdrawal speed of 2.1 mm/s and an intermediate soaking time of approximately 30 s.
  ○ After dip-coating, the samples are slightly dried in the air and then heated at 100 °C for approximately 20 h in clean air furnace.
  ○ After thermal treatment, the samples are cooled to room temperature at very slow cooling rate (furnace cooling) to obtain coatings of uniform thickness.

Table 4 presents the thicknesses of the coatings obtained by using different concentrations (wt.%) of UHMWPE polymer powder in decalin solution for the dip-coating process.

#### 2.1.6. Development of an Alternative Pre-Treatment Process for Si Substrates for Better Adhesion

Samad et al. [58] proposed an air-plasma treatment [59], which is an environmental friendly, cost effective, time effective and efficient pre-treatment process for Si substrates to improve the tribological properties of UHMWPE coatings. They reported a significant improvement in the tribological performance of the UHMWPE coatings deposited on the Si substrates after air-plasma treatment as compared to the substrates, which were subjected to piranha treatment. They found that the air-plasma treatment reduced the water contact angle (increased surface energy/wettability) of the Si substrate from 38.4° to 4.3° as compared to the piranha treatment, which reduced it to 21.3°. They reported an increase in the adhesion strength by a magnitude of two times and the wear life by a magnitude of 25 times for the UHMWPE coatings after the air-plasma treatment. Table 5 summarizes their results.

They attributed the effectiveness of the air-plasma treatment to its carbon-cleaning effect and oxidizing effect. As can be seen from the XPS results of Table 5, after the air-plasma treatment there was a reduction in the carbon content and an increase in the oxygen content. An increase in the oxygen content is observed after the air-plasma treatment because it generates functional groups (hydroxyl and carboxyl groups) on the Si substrate, which helps in increasing the wettability/surface free energy leading to an increase in the adhesion and the wear life of the coatings.

**Figure 8:**
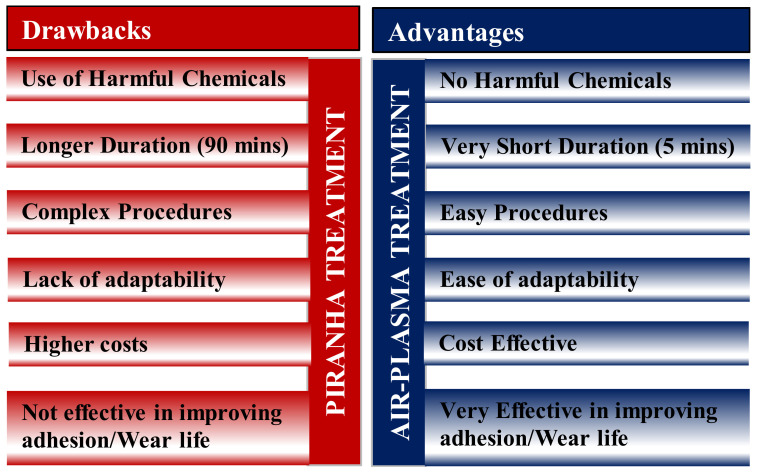
Drawbacks of the Piranha Treatment as compared to the Advantages of the Air-Plasma Treatment.

The air-plasma procedure was as follows: Si substrates were thoroughly cleaned using acetone and dried before they were placed in a Harrick Plasma Cleaner/Sterilizer under vacuum at an RF power of 30 W for approximately 5 min. Figure 8, shows a comparison of the piranha and the air-plasma treatments.

### 2.2. UHMWPE Coatings for Mechanical Bearing Applications

In the recent past, challenges such as global warming and energy conservation have motivated researchers to explore alternative lubrication strategies, which are more environmentally friendly and are effective in reducing the wear and friction between the various sliding components. The present-day lubrication strategies in mechanical components include the use of hard coatings or lubricants with additives. However, both strategies suffer from drawbacks. The hard coatings suffer from limitations such as a lack of chemical inertness to lubricants, poor adhesion to substrates, high deposition costs, complex deposition procedures etc. The strategy of using lubricants with additives also has its own set of problems such as, use of toxic chemicals, which are a potential health hazard, and which make it environmentally unfriendly. To overcome these challenges, polymer soft coatings are being investigated to act as protective coatings in mechanical components such as bearings, gears, cams, etc. Polymeric coatings have numerous advantages such as low friction, low wear, better adhesion, low depositional coats, simple depositional techniques etc. With this in mind, many research groups have explored the feasibility of using UHMWPE coatings as a protective layer in mechanical sliding systems. Researchers have found that UHMWPE because of its unique properties such as, low friction, high wear resistance, low moisture absorption and high impact resistance as already mentioned in the previous sections is a very potential candidate to be used as protective coatings in these sliding systems. However, to be used in these demanding applications, the load bearing capacity of the UHMWPE coatings has to be improved significantly. Researchers have taken different approaches to make the UHMWPE coatings suitable for such demanding applications. One of the approaches was to improve the adhesion between the coating and the substrate leading to better tribological performance. A second approach used was to fabricate nanocomposite and hybrid nanocomposite UHMWPE coatings by reinforcing UHMWPE with different nanofillers. Moreover, for the bearing applications, the usual substrates selected to deposit and test the coatings were DF3 Tool Steel, AISI 52100 bearing steel, stainless steel and aluminum. All these approaches will be discussed in detail in the following sections.

#### 2.2.1. Approach 1: Improving the Adhesion between the UHMWPE Coating and the Substrate Along with a PFPE Lubricant Overcoat

**Figure 9:**
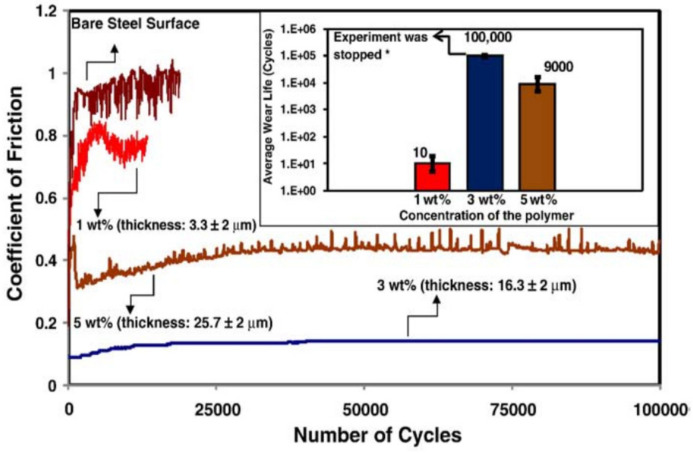
Typical coefficient of friction plots as a function of sliding cycles for different wt.% (different thicknesses) of UHMWPE at a normal load of 0.3 N and a rotational speed of 200 rpm. Inset: Average wear life for the three different thicknesses of the film [60]. Reproduced with permission from M. Abdul Samad, Nalam Satyanarayana, Sujeet K. Sinha, Surface and Coatings Technology; published by Elsevier, 2010.

Samad et al. [60], dip-coated UHMWPE coatings using different concentrations (1, 3 and 5 wt.%) on air-plasma treated DF3 tool steel (TS) samples to obtain different thicknesses (3.3, 16.3 and 25.7 µm), respectively. They evaluated the effect of thickness, normal load (0.3, 1, 2, 4 N) and rotational speed (200, 400, 600, 1000, 2000 rpm) on the tribological properties of TS/UHMWPE and TS/UHMWPE/PFPE using a ball-on-disk wear test. They observed that the UHMWPE coating deposited on the air-plasma treated TS samples showed a wear life of about 12 times greater than the coating deposited on the untreated TS samples. They attributed this to the effective carbon cleaning effect and the oxidizing effect of the air-plasma treatment, which helps in improving the adhesion between the coating and the substrate. They also found that the UHMWPE coating with an optimal thickness of 16.3 µm, resulted in a higher wear life (>100,000 cycles) as compared to the other thicknesses. They attributed this to the incomplete coverage of the substrate due to less amount of UHMWPE in case of lower thickness and a lump transfer of UHMWPE to the counterface in the initial stages of sliding leading to greater adhesion in case of thicker coatings. Figure 9 shows the effect of thickness on the tribological properties.

Their evaluation of the TS/UHMWPE coating at different loads and speeds, showed that the UHMWPE coating deposited on air-plasma treated TS samples had a PV factor (contact pressure X sliding velocity) of 94.6 MPa m/s. Furthermore, the dual coating of TS/UHMWPE/PFPE showed an extended wear life of about 130,000 cycles as compared to 700 cycles of TS/UHMWPE coating at a load of 4 N and a rotational speed of 2000 rpm. They attributed these improvements to the enhanced adhesion of the coating due to the air-plasma treatment and the lubricious property of the dual UHMWPE/PFPE coating.

#### 2.2.2. Approach 2: Improving the Load Bearing Capacity of the UHMWPE Coating by Reinforcing It with Nanofillers to Fabricate Nancomposite Coatings

One of the few approaches taken up by researchers to improve the load bearing capacity of the UHMWPE coatings to make them suitable for demanding tribological applications, was to fabricate UHMWPE nanocomposite coatings by reinforcing it with different nanofillers. A range of nanofillers such as, carbon nanotubes, nanoclay, alumina, graphene nanosheets, graphene nano platelets have been used and the effect of each of these fillers on the tribological properties of the UHMWPE coating will be discussed in this section.

Samad et al. [61,62,63,64] were instrumental in initiating the idea of developing UHMWPE nanocomposite coatings specifically for bearing applications. UHMWPE was reinforced with different concentrations (0.05, 0.1 and 0.2 wt.%) of carbon nanotubes (CNTs), dip-coated on air-plasma treated steel [61,62,63], and aluminum [64] substrates to form uniform nanocomposite coatings, which were tested under dry and base oil lubricated conditions [61,64]. Mechanical, thermal and tribological properties of the nanocomposite coating were evaluated [61]. They observed a significant improvement in the mechanical and thermal properties such as, hardness (Increase of 66%), elastic modulus (Increase of 58%) and thermal conductivity (Increase by 75%) of the nancomposite coating with an increase in the concentration of CNTs reinforcement (0.2 wt.%) as compared to the pristine UHMWPE coating. They attributed this improvement to the excellent inherent properties of CNTs and the uniform dispersion of CNTs in the UHMWPE polymer matrix. Ball-on-disk wear tests were conducted at a constant load of 4 N with varying speeds of 1000, 2000 and 2500 rpm. Nanocomposite coatings reinforced with 0.1 and 0.2 wt.% of CNTs did not fail, even until 10 million cycles at the highest speed of 2500 rpm, as compared to the wear life of 150,000 cycles for the pristine UHMWPE coating at a lower load of 2000 rpm. However, a slight increase in coefficient of friction from 0.08 to 0.16 was observed with the addition of CNTs, which was attributed to the increased hardness [61]. Table 6 presents the surface, mechanical and thermal properties of the UHMWPE nanocomposite coating reinforced with 0.1 wt.% of CNTs.

Samad et al. [62], also reported the effect of UV-radiation and different counterface materials (silicon nitride, AISI 52100 bearing steel and brass) on the triblogical properties of the abovedeveloped nanocomposite coating. Ball-on-disk wear tests were conducted using a 4 mm diameter ball made up of different materials as normal load of 4 N and a sliding speed of 2000 rpm. They found that the wear life of the nanocomposite coating was not affected by the change in the counterface material. However, brass showed a lower coefficient of friction as compared to silicon nitride and steel. Furthermore, the wear life of the nanocomposite coating did not alter even after a prolonged exposure to UV-radiation at an intensity of 0.71 W/m^2^ nm for 300 h.

Samad et al. [63], further dip-coated the developed nanocomposite coating on cylindrical steel shafts and evaluated the tribological properties of the same using a flat-on-cylinder configuration to simulate the real contact conditions (line contact) in a journal bearing. The coating was tested under different loads under dry and base oil lubricated conditions at room and elevated temperatures. To, further improve the tribological performance of the nanocomposite coating, an overcoat of PFPE was deposited, which reduced the coefficient of friction from 0.14 to 0.09. Moreover, the nanocomposite coating with/without the PFPE overcoat did not fail at elevated temperatures of 80 and 120 °C even after a test of 50 h, under dry conditions at a normal load of 60 N and a sliding speed of 0.11 m/s. This was attributed to the excellent thermal conductivity of the coatings due to the presence of CNTs. However, under base oil lubricated conditions, the UHMWPE+CNTs/PFPE coating could sustain a maximum temperature of 105 °C after, which a considerable amount of polymer softening was observed leading to the failure of the coatings.

Samad el al. [64] dip-coated the nanocomposite coating on aluminum substrates to evaluate the effect of substrate on the tribological properties under dry and base oil conditions. They observed that even when coated on aluminum substrates there was no deterioration in the wear life of the nanocomposite coating, which did not fail until 2 million cycles corresponding to a test duration of 100 h. Moreover, no softening of the coating was reported under base oil lubricated conditions at room temperature after a test duration of 100 h at a normal load of 60 N and a sliding speed of 0.11 m/s.

Han et al. [65] flame-sprayed UHMWPE nanocomposite coatings reinforced with different concentrations (0.15, 0.3 and 1 wt.%) of graphene nanosheets (GN) on grit blasted 304 L stainless steel substrates. Tribological properties were evaluated using a ball-on-disk reciprocating configuration at a constant load of 35 N and a sliding speed of 0.01 m/s. They reported a significant reduction in wear rate and coefficient of friction of about 20% and 25% respectively for the UHMWPE/1 wt.% GN coatings as compared to the pristine UHMWPE coatings. The improvement was attributed to an increase in the thermal stability and microhardness of the coatings. Electrochemical characterization of the UHMWPE/GN nancomposite coatings were also conducted and a significant improvement in the anti-corrosion properties are reported.

Samad et al. [66,67] dip-coated UHMWPE and UHMWPE/CNTs nancomposite coatings on air-plasma treated polyether ether ketone (PEEK) substrates. Even though, PEEK is a high-performance polymer because of its higher temperature resistance, it shows a higher wear rate and coefficient of friction (~0.3), which makes it a poor candidate for tribological applications. Hence, PEEK was surface modified with UHMWPE coatings [66] to improve its tribological performance. Ball-on-disk wear tests were conducted using a 440C stainless steel ball, at different normal loads (5, 7 and 9 N) and varying sliding speeds (0.1, 0.2 and 0.5 m/s). The UHMWPE coating (thickness = 27 ± 2 µm) was very effective in reducing the coefficient of friction rom 0.3 (uncoated PEEK) to 0.09 and also showed an excellent wear life of 250,000 cycles at a load of 7 N and a speed of 0.1 m/s. The improvement is attributed to the enhanced adhesion between the UHMWPE coating and the PEEK substrate because of the air-plasma pre-treatment, which helped in increasing the wettability of the surface (change in WCA: Untreated −93° to air plasma treated −32°) and to the excellent tribological properties of UHMWPE. However, it was reported that the UHMWPE coating could not sustain a higher load of 9 N. Hence, to further improve the load bearing capacity, air-plasma treated PEEK substrates were dip-coated with UHMWPE/CNTs nancomposite coatings with different concentrations (0.1 and 0.2 wt.%) of CNTs [67]. Two different concentrations (3 and 5 wt.%) of the UHMWPE polymer matrix were used to obtain different thicknesses of coatings. Among all the combinations tested, 3 wt.% UHMWPE/0.2 wt.% CNTs nanocomposite coatings showed a wear life of greater than 25,000 cycles at a higher load of 9 N and a sliding speed of 0.2 m/s. This improvement in the load bearing capacity of the nanocomposite coatings is attributed to the uniform dispersion of CNTs in the polymer matrix and the improvement in the mechanical properties of UHMWPE due to the addition of CNTs.

Azam et al. [68], reinforced UHMWPE with different concentrations of nanoclay (0.5, 1.5 and 3 wt.%) and deposited the nanocomposite coatings (thickness = 125 µm) on air-plasma-treated aluminum substrates using an electrostatic powder spraying technique. Nanoclay was used for its excellent mechanical and barrier properties. Ball-on-disk wear tests using a 440C stainless steel ball were conducted at different loads and sliding speeds (0.1, 0.2 and 0.3 m/s) to evaluate the wear life and friction properties of the optimized coating with the optimized loading of nanoclay. It was reported that UHMWPE reinforced with 1.5 wt.% of nanoclay exhibited the best wear performance as the coating as it did not fail even until 100,000 cycles (sliding distance of 1.3 km) at a normal load of 9 N and a sliding speed of 0.1 m/s. The improvement was attributed to the uniform dispersion of nanoclay within the UHWMWPE polymer matrix resulting in an exfoliated morphology. The developed nanocomposite coating has the advantage of an increased load bearing capacity with the advantages such as low cost, ease of fabrication, low friction and low wear which makes it an excellent candidate to be used as protective and wear resistant coating in demanding tribological applications such as bearings.

Ismaila et al. [69,70,71], used different concentrations (0.25, 1 and 2 wt.%) of graphene nanoplatelets (GNPs) to reinforce UHMWPE and deposited the nanocomposite coating on air plasma treated aluminum substrates using electrostatic spraying technique. Pin-on-disk wear tests were conducted using a hardened tool steel pin to optimize the loading of GNPs. They reported that UHMWPE reinforced with 1 wt.% of GNPs resulted in a 51% reduction in wear as compared to the pristine UHMWPE coating. The optimized coating was further tested at different loads and different sliding speeds to determine the operating limits of the coating, which was found to be 4 MPa m/s [69]. Further to this study, Ismaila et al. [70] proceeded to coat aluminum thrust bearings in the form of a ring, to simulate the actual conditions and tested the coatings using a ring-on-disk tests against a steel disk. The tests were carried out under dry and base oil lubricated conditions. They reported an increase of 440% in the load bearing capacity of the UHMWPE/1wt.% GNPs nanocomposite coating under the presence of base oil. Ismaila et al. [71], also investigated the effectiveness of the developed UHMWPE/GNPs nanocomposite coating for its anti-corrosion performance by coating it on an aluminum alloy substrate. They found that UHMWPE/2wt.% GNPs was the most efficient nanocomposite coating in providing the highest corrosion resistance in 3.5% NaCl solution.

Samad [72], was able to increase the load bearing capacity of the UHMWPE coatings to as high as 12 N by reinforcing it with alumina (Al_2_O_3_) nanoparticles. Different concentrations (0.5, 3, 5 and 10 wt.%) of alumina were used to form UHMWPE nanocomposite coatings (thickness = 60 ± 3 µm), which were electrostatically deposited on air-plasma steel substrates. Ball-on-disk wear tests were conducted using a 440C stainless steel ball at different loads (7, 9 and 12 N) and at a sliding speed of 0.1 m/s. It was reported that the UHMWPE nanocomposite coating reinforced with 3 wt.% and 5 wt.% of alumina nanoparticles did not fail even until 250,000 cycles at a load of 12 N, whereas the pristine UHMWPE coating failed at a lower load of 9 N at the same sliding speed of 0.1 m/s. This increase was attributed to the improvement in the hardness of the coating with the addition of hard alumina nanoparticles and their uniform dispersion in the UHMWPE polymer matrix. The predominant wear mechanisms for the composite coatings were found to be a combination of abrasive and adhesive wear.

Guo et al. [73], used a new method of electrophoric deposition of UHMWPE particles in combination of an electroplating of nickel (Ni) to form Ni/UHMWPE composite coatings on copper substrates. They also deposited Ni/UHMWPE composite coatings using the traditional electroplating method and compared the tribological performance of both the coatings. A ball-on-disk configuration with a 304 stainless steel ball as a counterface was used at a load of 2 N and a sliding speed of 0.1 m/s. They found that the tribological performance of the Ni/UHMWPE composite coating deposited by the electrophoric deposition method performed excellently well as compared to the traditional electroplating method. It showed a low coefficient of friction of ~0.2 to 0.3, a 57% reduction as compared to the pristine Ni coating.

Akeem et al. [74], developed UHMWPE composite coatings by reinforcing it with different concentrations (1, 3, 6 and 9 wt.%) submicron tungsten carbide (WC) particles and electrostatically sprayed the coatings on air-plasma treated steel substrates. They reported an increase in the mechanical properties and the adhesive strength with the substrates with an increase in the WC content until 6 wt.%. However, these properties were reduced significantly as the WC content increased, which was attributed to the non-uniform dispersion of the WC particles tending to form agglomerations. They conducted potentiodynamic polarization and electrochemical characterizations of the developed nanocomposite coatings and found that UHMWPE/1 wt.% WC nanocomposite coating exhibited the highest corrosion resistance due to the uniform dispersion of the WC particles in the UHMWPE polymer matrix. An increase of 80% in corrosion resistance was observed for the nanocomposite coatings as compared to that of the pristine UHMWPE.

#### 2.2.3. Approach 3: Improving the Tribological Performance of the Uhmwpe Coating by Reinforcing It with More Than One Nanofiller to Fabricate Hybrid Nanocomposite Coatings

In the recent past, a great deal of interest has been generated by an emerging approach, which is the fabrication of hybrid nanocomposite coatings. Researchers are extensively exploring this new approach of fabricating hybrid nanocomposite coatings by reinforcing UHMWPE with more than one filler, to take advantage of the individual properties of each of the filler into one product. However, the selection of the nanofillers becomes very significant depending upon the targeted application.

**Figure 10:**
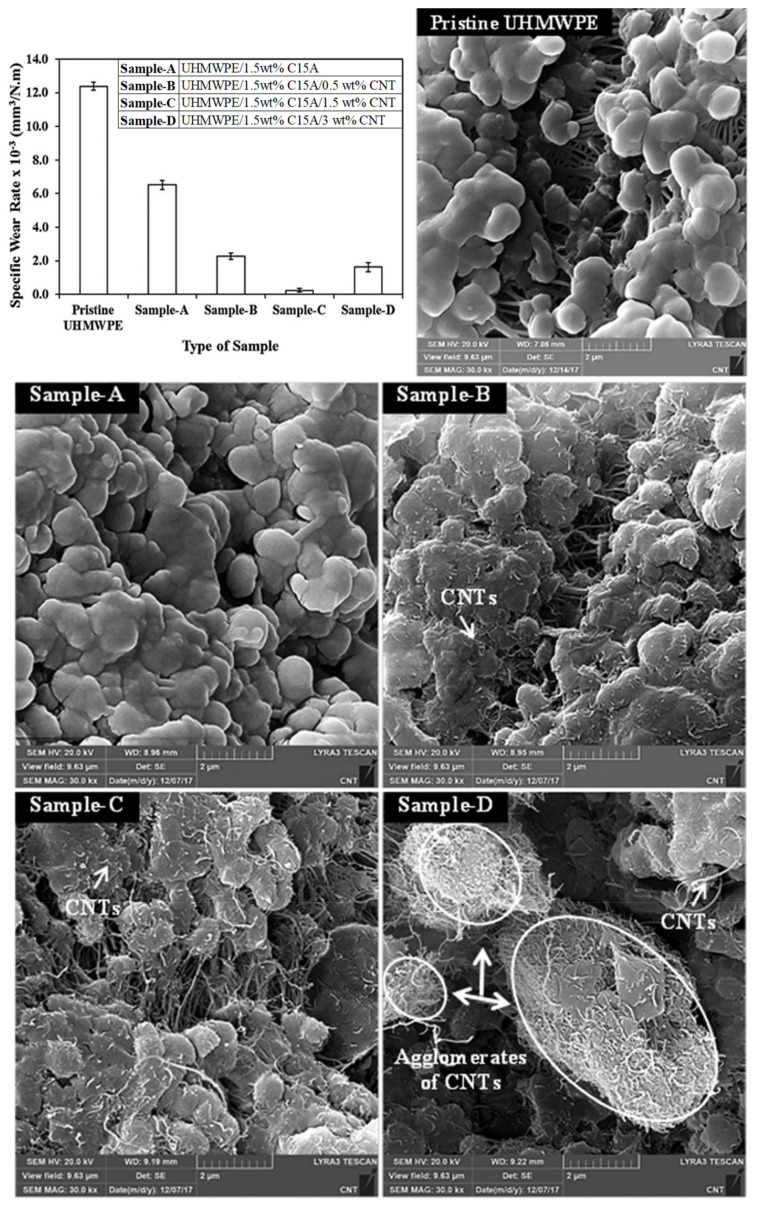
Comparison of specific wear rates of the different coatings at a load of 12 N and a sliding speed of 0.1 m/s. SEM Micrographs of different samples showing the dispersion [75]. Reproduced with permission from Azam, M.U.; Samad, M.A, Journal of Tribology; published by ASME, 2018.

Azam et al. [75] conducted one such study whereby they developed a novel hybrid nanocomposite coating of UHMWPE reinforced with two nanofillers, namely: nanoclay (C15A) and carbon nanotubes (CNTs). They selected nanoclay based upon its excellent mechanical properties and barrier properties. CNTs were selected because of their outstanding thermal and mechanical properties. The hybrid nanocomposite coating was fabricated by keeping the concentration of the nanoclay as 1.5 wt.%, which was found to be the optimum loading from a previous study [68]. Different concentrations (0.5, 1.5 and 3 wt.%) of CNTs were used to fabricate the hybrid nanocomposite coatings powder, which were electrostatically deposited (thickness = 180 µm) on air-plasma treated aluminum substrates. Ball-on-disk wear tests were conducted using a 440C stainless steel ball under dry conditions at different normal loads and sliding speeds. As expected, the hardness of the hybrid nanocomposite coatings increased with an increase in the CNT content. The wear tests revealed that the load bearing capacity of the pristine UHMWPE coating was 7 N, which was improved to 9 N after the addition of 15. wt.% of nanoclay alone, which was further increased to 12 N with the addition of 1.5 wt.% of CNTs. The hybrid nanocomposite coating UHMWPE/1.5 wt.% C15A/1.5 wt.% CNT showed an excellent wear resistance as shown in Figure 10, which is attributed to the uniform dispersion of CNTs within the UHMWPE matrix. The UHMWPE/1.5 wt.% C15A/1.5 wt.% CNT showed excellent wear resistance, even at increased speeds of 0.2 m/s.

**Figure 11:**
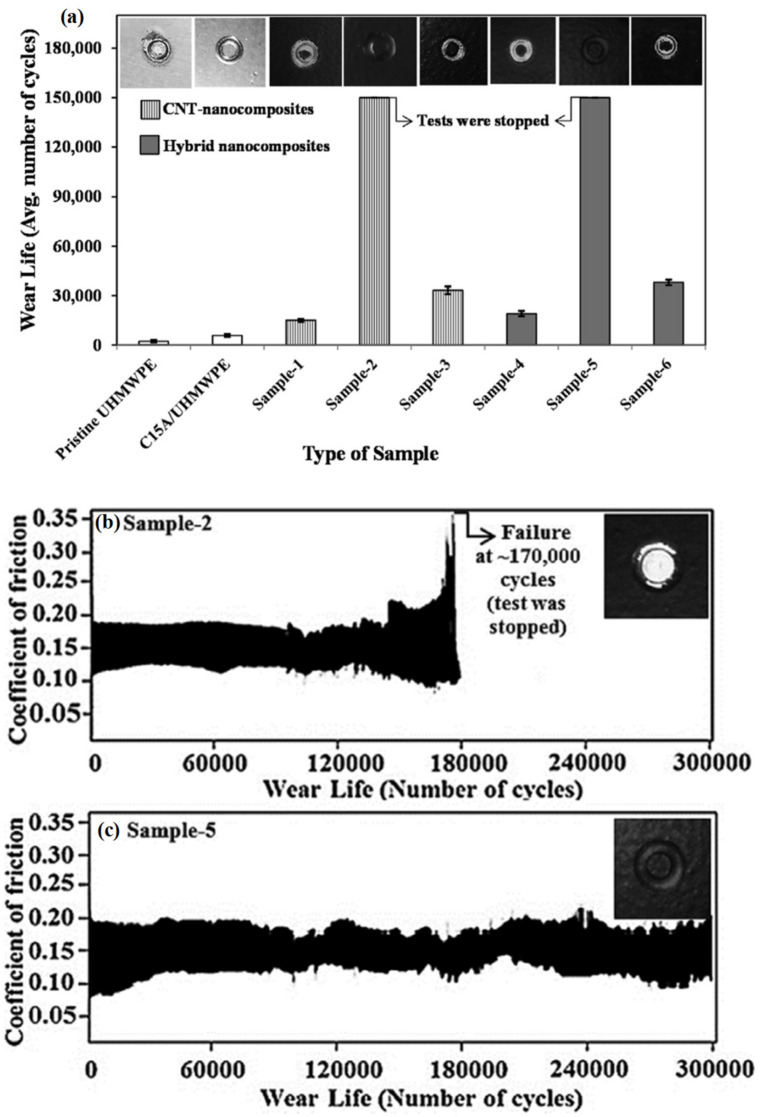
(**a**) Comparison of wear lives of different UHMWPE coatings: Sample-1 = UHMWPE/0.5 wt.% CNTs, Sample-2 = UHMWPE/1.5 wt.% CNTs, Sample-3 = UHMWPE/3wt.% CNTs, Sample-4 = UHMWPE/1.5 wt.% C15A/0.5 wt.% CNTs, Sample-5 = UHMWPE/1.5 wt.% C15A/1.5 wt.% CNTs, Sample-6 = UHMWPE/1.5 wt.% C15A/3 wt.% CNTs for 150,000 cycles. (**b**) Wear life of Sample-2 for a test duration of 300,000 cycles. (**c**) Wear life of Sample-5 for a test duration of 300,000 cycles. All the tests conducted at a normal load of 12 N and a sliding seed of 0.1 m/s under water [76]. Reproduced with permission from Azam, M.U.; Samad, M.A, Tribology International; published by Elsevier, 2018.

Azam et al. [76], further investigated the tribological performance of the developed hybrid nanocomposite coatings under water without and with abrasives. One of the major advantages of nanoclays is their platelet-like structure resulting in their excellent barrier properties, which makes them an ideal reinforcement to improve the water or any liquid uptake resistance of polymers. Hence, ball-on-disk wear tests were conducted under water without abrasives to evaluate the tribological performance of the hybrid nanocomposite coatings. Figure 11 shows the wear life of different samples tested under water, at a load of 12 N and at a sliding speed of 0.1 m/s. Initially, both, UHMWPE/1.5 wt.% CNTs and UHMWPE/1.5 wt.% C15A/1.5wt.% CNTs nanocomposite coatings showed a wear life of about 150,000 cycles under water. But as the test duration was increased to 300,000 cycles, only the hybrid nanocomposite coating of UHMWPE/1.5 wt.% C15A/1.5 wt.% CNTs passed the test indicating the excellent barrier properties of nanoclay C15A, leading to an increased resistance to water uptake at higher loads. Wear tests were also conducted on the best hybrid nanocomposite coating of UHMWPE/1.5 wt.% C15A/1.5 wt.% CNTs, in the presence of abrasives dispersed in water at a load of 12 N and a sliding speed of 0.1 m/s. An increase in the wear resistance of the hybrid nanocomposite coating was reported in the presence of abrasives and this was attributed to the property of the soft polymer matrix, which helps in embedding the hard particles leading to the strength of the coating.

#### 2.2.4. Summary

**Figure 12:**
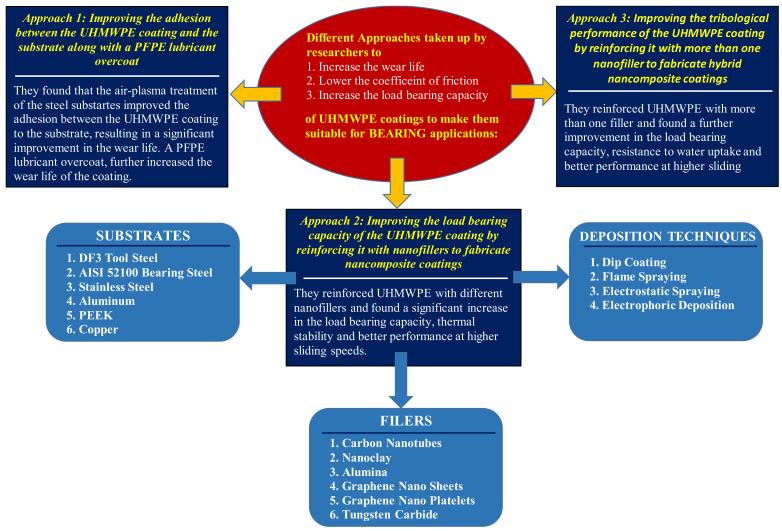
Schematic of the different approaches used by researchers to make the UHMWPE coatings suitable for demanding tribological applications such as Mechanical Bearings. It also lists out the different substrates, nanofillers and the deposition techniques used for fabricating the nanocomposite coatings.

Figure 12, shows the summary of all the approaches taken up by the researchers to make the UHMWPE coatings suitable for demanding tribological applications such as mechanical bearings. It also shows the different nanofillers that have been used to reinforce the UHMWPE coating and lists out the different deposition techniques.

##### Coating Deposition Techniques

In view of the extensive literature reviewed, we can short list the various coating deposition techniques used to fabricate the UHMWPE coatings on a variety of substrates. These techniques are explained in detail below.

**Figure 13:**
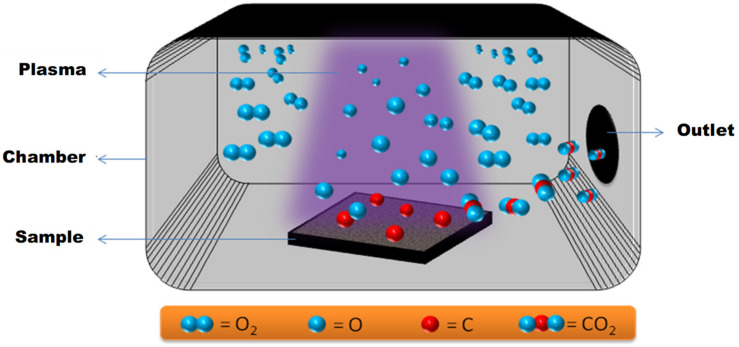
Schematic of the Air-Plasma Treatment showing the Carbon Cleaning Effect and the Oxidizing effect.

**Figure 14:**
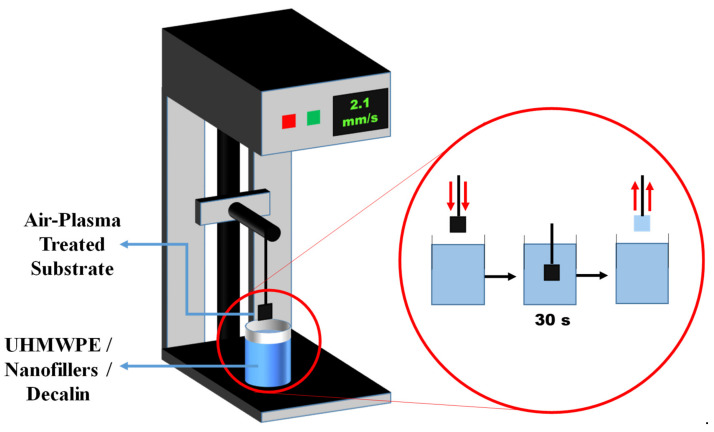
Schematic showing the complete dip coating process.

**Figure 15:**
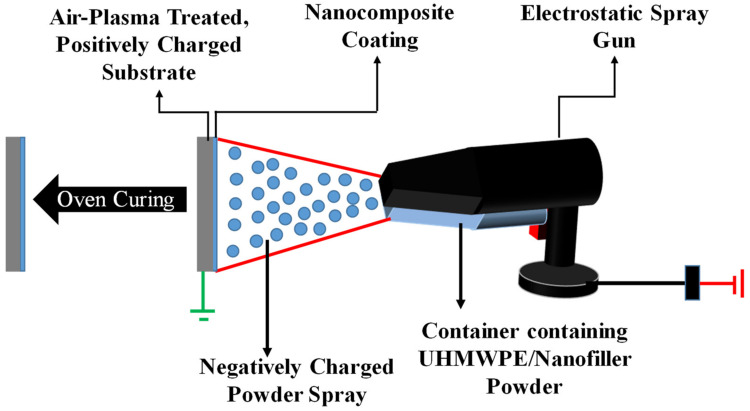
Schematic of the Electrostatic Spraying Technique.

**Figure 16:**
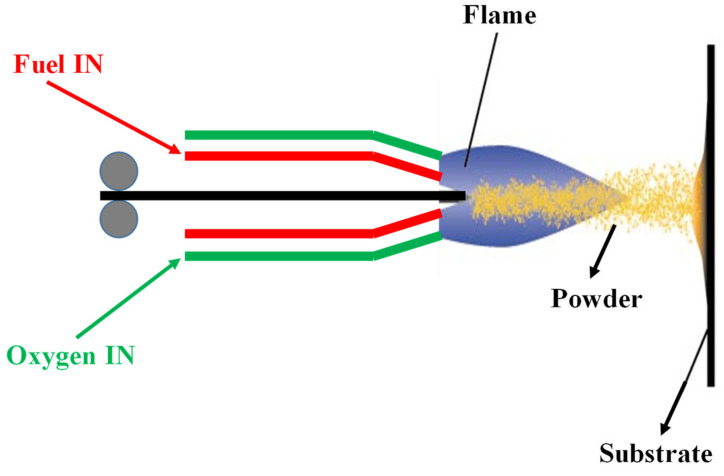
Schematic of the Flame Spraying Technique.

**Figure 17:**
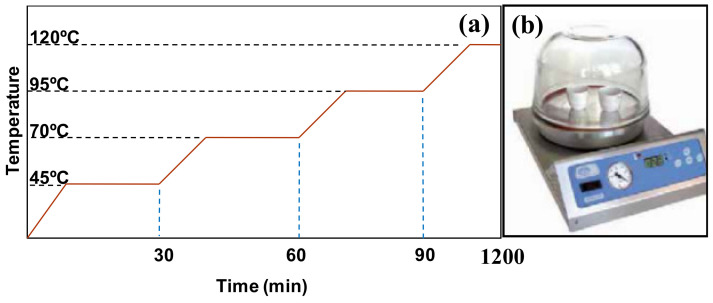
(**a**) Schematic of the stepwise post-heat treatment used for the consolidation of the UHMWPE nanocomposite coatings [60,61,62,63,64,66,67]. (**b**) The heating plate used for the post-heat treatment process.

It is to be noted that, all the researchers used the following steps in the overall coating procedure:

- *Cleaning of the substrate*: The substrates are cleaned thoroughly to make the surfaces devoid of any grease, oil, hydrocarbons and impurities to improve the adhesion with the coating. In all the cases presented, the substrates were cleaned with acetone or ethanol and then drying it with dry air or nitrogen.
- *Pre-treatment of the substrate*: Pre-treatment of the substrate is one of the very important steps in the coating procedure as it helps in improving the adhesion of the coating to the substrates, which in turns effects its tribological performance.
- *Air-Plasma Treatment*: The most favorable pre-treatment procedure in the deposition of the UHMWPE and its composite coatings has been found to be air-plasma treatment, irrespective of the substrate material. Substrates were placed in a Harrick Plasma Cleaner/Sterilizer under vacuum at an RF power of 30 W for approximately 5 min. Air-plasma treatment has been found to be an effective pre-treatment method, which is environmental friendly, easy to use, very flexible/adaptive, cost effective and less time consuming [60,61,62,63,64,66,67,68,69,70,71,72]. Air-plasma treatment helps in cleaning the surface by carbon cleaning effect and at the same time functionalizes the surface with carboxyl and hydroxyl groups which in turn increases the wettablility/surface free energy of the surface leading to an improved adhesion between the coating and the substrate. Figure 13 shows a schematic of the air-plasma treatment.

- *Grid blasting*: Han et al. [65] used grid blasting followed by pre-heating the substrate to a temperature of 110–130 °C.
- *Preparing the composite powders solution or dry powders by using different dispersion techniques*: Dispersion of the nanofillers is a crucial step in the preparation of nanocomposite coatings as it determines the final mechanical properties of the prepared coatings. Researchers have used different techniques to disperse the nanofillers uniformly in the UHMWPE polymer matrix depending upon the coating technique used. The various techniques used are explained below.
- Nanopowder solution from dip-coating: Dip-coating has been used by [60,61,62,63,64,66,67] to coat steel, aluminum and PEEK substrates with UHMWPE and UHMWPE composite coatings reinforced with CNTs. The nanopowder solution for dip coating was prepared by plasma treating the CNTs in the Harrick Plasma Cleaner/Sterilizer by uniformly spreading it out in a flat dish. The required amount of plasma-treated CNTs were then subsequently added to a measured quantity of decahydronapthalene (decalin), which is the solvent used to dissolve UHMWPE polymer. The CNTs were sonicated for 12 min using a probe sonicator with an amplitude of 30% and a cycle on/off time of 20/5 s to ensure uniform dispersion of CNTs in decalin. Required amount of UHMWPE depending upon the concentration needed, is added to the mixture of CNTs and decalin and the whole mixture is subjected to magnetic stirring for 20 min to ensure proper mixing of CNTs and UHMWPE. The mixture is then heated to a temperature of 170 °C for 1 h to completely dissolve the UHMWPE powder. Magnetic stirring is used throughout the heating process for uniform heat distribution, which aids in the complete dissolution of the UHMWPE powder.
- *Nanopowders for electrostatic spray coating*: The electrostatic spray coating technique has been used by [68,69,70,71,72], to deposit the coatings on steel and aluminum substrates. The nanopowders were prepared by ultrasonicating the required amount of fillers in 50 mL of ethanol using a probe sonicator with an amplitude of 30% and a cycle on/off time of 20/5 s for 10 min followed by magnetic stirring for 2 min to ensure uniform dispersion. Weighted amount of UHMWPE powder is then added to the solution and subjected to magnetic stirring for another 60 min. The UHMWPE/filler solution is then subsequently heated to a temperature of 70 °C on a heating plate for 24 h to ensure the complete evaporation of ethanol leaving behind dry nanopowder.
- *Coating deposition techniques*: In the literature, four different techniques have been used to deposit UHMWPE composite coatings on a variety of substrates. Selection of proper coating technique is a significant step to ensure uniform coating thickness without any defects. The procedures for various techniques used are explained below:
- *Dip coating process*: Dip coating was found to be a very simple, cost effective and efficient technique to coat UHMWPE composite coatings. One of the advantages of dip coating is its, easy adaptability and ease of coating any intricate shapes irrespective of the substrate material [60,61,62,63,64,66,67]. The process for preparing the solution of UHMWPE/fillers in the decalin solvent has been specified above. For the dip coating, the pre-treated substrates are dip coated using a simple speed controlled dip coating machine. An optimum dipping speed and the withdrawal speed of 2.1 mm/s was used to obtain coatings of uniform thickness. The dipping time of the substrate in the solution was maintained at 30 s. Figure 14 shows the schematic of the complete dip coating process.

- *Electrostatic spray coating process*: Electrostatic spraying is excellently suitable for conductive materials. It’s a very efficient, cost effective, simple and a flexible/adaptive technique, used by researchers to deposit UHMWPE coatings on metallic substrates [68,69,70,71,72]. The basic principle of electrostatic spray gun is that negatively charged powder particles are deposited on positively charged substrate. For this process, the air-plasma treated substrates were pre-heated for 5 min to a temperature of 180 °C on a heating plate and then the nanopowders were sprayed using an electrostatic gun, which charges the powder particles and deposits the powder on the charged substrate. Figure 15 shows the schematic of the electrostatic spraying technique.

- *Flame spraying technique*: A flame spraying technique was used to deposit UHMWPE nanocomposite coatings reinforced with graphene nanosheets [65]. The flame spraying technique is a cost-effective method of depositing UHMWPE coatings with good adhesion and mechanical properties on a variety of substrates [65]. Figure 16 shows a schematic explaining the flame spraying technique. Han et al. flame sprayed UHMWPE/GN nanocomposite powder using a FS-4 multifunctional powder flame spray torch and employed acetylene as the fuel gas and oxygen as the oxidant. The pressure and flow rate of oxygen and acetylene were fixed at 0.3 MPa, 0.25 m^3^/h, and 0.1 MPa, 0.30 m^3^/h, respectively. Compressed air was also used for the spraying, and the pressure and flow rate were 0.4 MPa and 3–5 m^3^/h, respectively. The powder feeding rate was 15 g/min, and the spray distance was 250 mm.

- *Post-heat treatment for the consolidation of the coating*: The post-treatment is the final and a very important step in total coating procedure as this step helps in the consolidation of the polymer coating. Based upon the different coating techniques used, researchers have used different post-heat treatment procedures, which are explained below.
- *Post-heat treatment for dip coated samples*: [60,61,62,63,64,66,67] used a unique stepwise post-heat treatment process for the proper consolidation of the polymer to obtain nanocomposite coatings with uniform thickness. Figure 17 shows the schematic of the stepwise heating process used. The coated samples were exposed to temperatures of 45 °C for 30 min, followed by 70 °C for another 30 min, followed by 95 °C for another 30 min and then left at a temperature of 120 °C for the remaining time of 20 h.

- *Post-heat treatment for electrostatic sprayed samples:* After the samples were electrostatically sprayed with the nanocomposite powders, the coated samples were post heat treated at a temperature of 1800 C on a heating plate for 35 min, which were then left to cool down to room temperature prior to further characterizations [68,69,70,71,72].
- *Post-heat treatment for flame sprayed samples*: No post-heat treatment process was used for the flame sprayed samples. The samples were just allowed to cool down to room temperature prior to further characterizations [65].

##### Different Characterization Techniques

**Figure 18:**
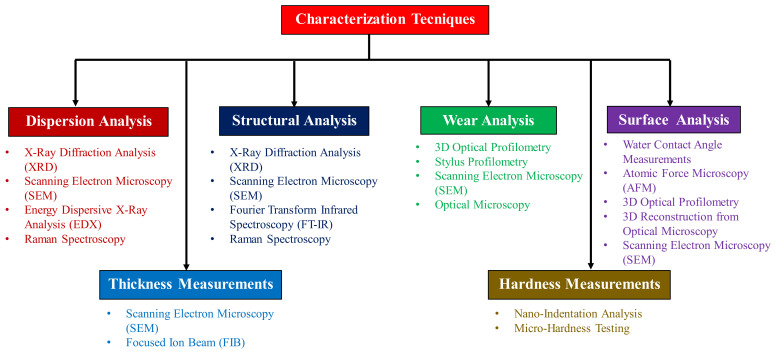
Various techniques used for characterizing the UHMWPE Nanocomposite coatings by different researchers.

As can be seen from the extensive review, researchers have used different characterization techniques to evaluate the surface and mechanical properties of the UHMWPE nanocomposite coatings. Figure 18 lists out the variety of characterization techniques used for different purposes.

- *X-Ray diffraction Analysis (XRD):* Researchers have extensively used this powerful technique to characterize the dispersion of different nanofillers such as CNTs and nanoclays within the UHMWPE matrix. Samad et al. [63] used it to evaluate any change in the crystallinity and degradation of UHMWPE coating on the addition of CNTs at different temperatures. They found that there was no significant shift in the signature peaks of UHMWPE at elevated temperatures and concluded that there was no change in the crystallinity of the UHMWPE/CNTs nanocomposite coating at elevated temperatures. Umar et al. [68] used the XRD analysis to study the dispersion of nanoclay in the UHMWPE matrix and to ascertain the developed morphology. The platelet-like structure of nanoclay when dispersed within the UHMWPE matrix, can result in an intercalated, phase separated or exfoliated morphologies. They qualitatively evaluated the developed morphology by studying the shifting of the signature XRD peaks of nanoclay. They found out that, UHMWPE/1.5 wt.% nanoclay resulted in an exfoliated morphology with uniform dispersion of nanoclay within the UHMWPE matrix.
- *Raman spectroscopy*: Researchers have also used Raman spectroscopy to ascertain the dispersion of the nanofillers and also to evaluate their interfacial interaction with the UHMWPE matrix. Umar et al. [75,76], used Raman spectroscopy to evaluate the interaction of CNTs with the UHMWPE matrix, based upon the shifting of the peaks corresponding to the G-band to higher wave numbers. The shifting of the G band towards higher frequencies is attributed to the disentanglement of the CNTs in the UHMWPE matrix as a result of polymer penetration into the CNT bundles, resulting in a good load transfer. Ismaila et al. [69], used Raman spectroscopy to evaluate the dispersion of GNPs within the UHMWPE matrix based upon the presence/absence of the signature peaks related to GNPs in the raman spectra.
- *Scanning electron microscopy (SEM)/energy dispersive X-Ray analysis (EDX):* One of the most common characterization technique used to quantitatively analyze the dispersion of the nanofillers within the UHMWPE matrix is SEM in conjunction with EDX which helps in obtaining the elemental mapping of the nanofillers [63,68,69,75,76]. The combination of these two techniques has also been used to ascertain the failure or the non-failure of the coatings by most of the researchers. The presence of a peak in the EDX spectrum corresponding to that of the substrate material would indicate the failure of the coating. SEM has also been used to determine the thickness of the coatings by looking at the cross-sectional view of the sample. Wear mechanisms that the coating has undergone and the transfer film phenomena have also been evaluated by examining the wear tracks using SEM/EDX.
- *3D-Optical profilometry***:** This is one of the most effective techniques used by the researchers to quantify the wear depth and the wear volume loss of the coating [61,62,63,64,65,66,67,68,69,70,71,72]. This also helps to determine the failure of the coating by comparing the wear depth to the thickness of the coating. Surface analysis such as, quantifying the surface roughness of the UHMWPE coatings has also been conducted by profilometry.

##### Different Configurations Used in Tribological Testing

**Figure 19:**
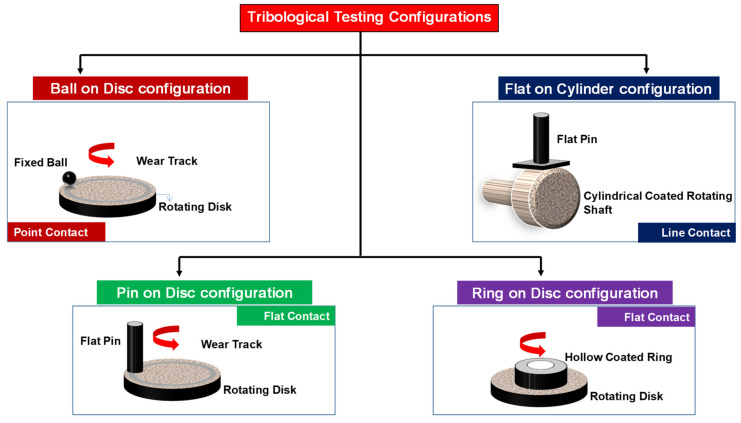
Different configurations used by researchers to conduct tribological characterizations of the UHMWPE nanocomposite coatings.

Researchers have used different configurations for the tribological testing of the UHMWPE and the UHMWPE composite and hybrid composite coatings. Figure 19, shows the different configurations used for the tribological testing of the UHMWPE coatings. The configurations were selected to simulate the actual operating conditions as much as possible.

- *Ball on disk (point contact)*: This configuration is the most predominantly used wear test configuration in most of the studies because of the high contact pressures it simulates due to the point contact [52,53,54,55,56,57,58,60,61,62,66,67,68,72,75,76]. This helps in testing the coating at very high contact pressures, which in turn helps in determining the PV limits of the coating. It is also to be noted that, the ball on disk configuration has a few disadvantages such as the changing pressure due to the change in the contact area over the time.
- *Flat on cylinder (line contact)*: Samad et al. [63,64] used the flat on cylinder configuration to simulate a line contact as in real journal bearings. However, the contact pressures resulting in this configuration are lower as compared to the ball on disk contact, due to an increase in the contact area.
- *Pin on disk (flat contact)*: Ismaila et al. [69,71] used the pin on disk configuration to simulate a mechanical bearing. The advantage of this configuration is that the contact pressure remains constant through out the wear test.
- *Ring on disk (flat contact)*: Ismaila et al. [70] used the ring on disk configuration to simulate a mechanical bearing in a more accurate manner under dry and boundary lubricated condition. The advantage of this configuration is that the contact pressure remains constant throughout the wear test.

### 2.3. UHMWPE Coatings for Biomedical Applications

UHMWPE, being a highly wear resistant polymer exhibiting low coefficient of friction, also is an excellent biocompatible polymer. Moreover, it also has the highest impact resistance among all the polymers, which makes it an excellent candidate for biomedical applications. Numerous attempts have been made to use pristine UHMWPE or UHMWPE composites in the bulk form in biomedical applications [77]. However, there are very few studies where efforts have been made to explore the feasibility of using UHMWPE in the form of coatings in biomedical applications. Traditionally, metals such as stainless steels, cobalt alloys, titanium alloys have been extensively used as biomaterials for applications such as joint replacements, dental roots, orthopedic fixation, and stents. However, these metallic biomaterials suffer from poor tribological performance such as moderately high friction and low wear resistance [78]. Several approaches have been explored by researchers to improve the tribological properties of the metallic biomaterials by fabricating composites, surface modifying them by microstructural changes and also by depositing biocompatible coatings [78]. With UHMWPE being a biocompatible material, a few studies have been conducted to develop UHMWPE coatings and hybrid nanocomposite coatings to surface modify titanium alloys in order to improve their tribological performance. The two approaches present in the literature are presented below.

#### 2.3.1. Approach 1: Improving the Tribological Properties of Titanium Alloys Using a UHMWPE Coating Along with a PFPE Lubricant Overcoat

Panjwani et al. [79] made one of the first attempts to develop UHMWPE coatings for biomedical applications. They dip-coated titanium alloy samples (Ti6Al4V), which were plasma pre-treated with pristine UHMWPE coatings of approximately 19.6 ± 2 µm thickness. Ball on disk configuration was used for conducting wear tests at different loads (0.5, 1, 2 and 4 N) and different rotational speeds (200 and 400 rpm) to evaluate the wear durability of the developed coatings. It was found that the pristine UHMWPE coating showed a low coefficient of friction of 0.1 and a wear life of greater than 175,000 cycles at a load of 4 N and a speed of 0.08 m/s. This was attributed to the strong adhesion between the coating and the titanium substrate and also to the excellent tribological characteristics of the UHMWPE coating. To, further improve the wear durability of the UHMWPE coating, an ultra-thin layer of PFPE lubricant, which is also a biocompatible lubricant, was coated on top of the UHMWPE coating, resulting in an increased wear life. This dual coating was tested at an increased speed of 1000 rpm and it showed a wear life of ~60,000 cycles as compared to ~28,000 cycles of the single UHMWPE coating. The developed UHMWPE coating also passed the cytotoxicity test performed by NAMSA (Northwood, OH, USA) according to the guidelines of International Organization for Standardization 10993-5: Biological Evaluation of Medical Devices, Part 5: Tests for In Vitro Cytotoxicity.

#### 2.3.2. Approach 2: Improving the Tribological Properties of Titanium Alloys Using a UHMWPE Hybrid Nanocomposite Coating

Zahid et al. [80], developed UHMWPE hybrid nanocomposite coatings by reinforcing them with two biocompatible nanofillers; carbon nanotubes (CNTs) and hydroxyapatite (HA). Different concentrations (0.5, 1.5 and 3 wt.%) of CNTs and different concentrations (0.5, 1.5, 3 and 5 wt.%) of HA were used to fabricate the hybrid coatings and deposit on air-plasma treated titanium (Ti6Al4V) substrates using the electrostatic spraying technique. Ball on disk tribological tests were conducted at different loads (7, 9, 12 and 15 N) at a constant sliding speed of 0.1 m/s. Initially, the loading of CNTs in UHMWPE was optimized, which was found to be 1.5 wt.%. Subsequently, the different loading of HA was added to the UHMWPE/1.5 wt.% CNTs and the best combination of CNTs and HA was determined. It was observed that, hybrid nanocomposite of UHMWPE/1.5 wt.% CNTs/3 wt.% HA showed an excellent wear life of greater than 250,000 cycles with a lower wear rate, at a load of 12 N and a sliding speed of 0.1 m/s. This was attributed to the improved adhesion between the hybrid nanocomposite coating to the substrate and also to the inherent good properties of both the filler materials, which helped in improving the hardness of the coating.

#### 2.3.3. Summary

**Figure 20:**
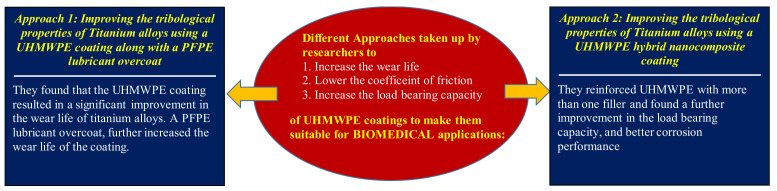
Schematic of the different approaches used by researchers to make the UHMWPE coatings suitable for biomedical applications.

Figure 20 shows a summary of all the approaches taken up by the researchers to make the UHMWPE coatings suitable for biomedical applications.

It should be noted that the different, coating techniques (dip coating and electrostatic spraying technique), pre-treatment techniques of the substrates (air-plasma treatment) that have been used to modify the titanium alloys have already been detailed in the above sections.

## 3. Overall Summary

Table 7, Table 8 and Table 9 give an overall summary of the types of coatings, dispersion techniques, operating conditions, (load, speed, temperature), coating thicknesses, coating techniques, wear life and coefficient of friction, types of nanofillers used etc. for all the developed UHMWPE and its composite coatings for the MEMS, bearing and bio-medical applications, respectively.

### A Few Observations

In this section, the various factors affecting the tribological behavior and the performance of the UHMWPE coatings in view of the extensive literature presented above are summarized. The different wear mechanisms observed for the UHMWPE coatings are also discussed elaborately.

- *Transfer Film Formation*: It is to be noted that one of the factors that plays a very significant role in the tribologial performance of the UHMWPE coatings is the formation of the transfer film on the counterface material. Various researchers have reported that this transfer film plays a significant role in enhancing the tribological properties of the UHMWPE coating based upon its adherence to the counterface material. It has been reported that the addition of nanofillers result in a transfer film with good adhesion with the counterface body, which helps in reducing the wear rate and the coefficient of friction of the UHMWPE coatings.
- *Nanofillers*: As can be seen from the extensive literature presented, the addition of nanofillers to the UHMWPE matrix helps in improving the wear resistance of the UHMWPE coatings. These nanofillers help in anchoring the long polymer chains by acting as bridges between them, which helps in improving the resistance to material pull-out leading to higher wear resistance with reduced wear debris formation.
- *Coating Adhesion*: One of the factors that significantly affects the tribological performance of the UHMWPE coating is its adhesion to the substrate. Researchers have used various routes to improve the adhesion as discussed earlier. Pretreatments such as, piranha treatment, air-plasma treatment and pre-heating have been used to improve the adhesion. Of all the pre-treatments, air-plasma treatment was found to be the most effective, environmentally friendly and flexible.
- *Wear mechanisms*: Most researchers have reported the failure mode of ultra-high molecular weight polyethylene coatings by a combination of two pre-dominant wear mechanisms; adhesive wear and abrasive wear. Adhesive wear is observed in the first few cycles of sliding because of the polymer lump transfer from the substrate to the counterface material. However, if the transfer film strongly adheres to the counterface material, it is a case of polymer on polymer sliding which helps in protecting the coating from getting exposed to the hard asperities of the counterface material resulting in reduced wear rates and reduced friction coefficients. However, if the adherence of the transfer film to the counterface material is weak, it results in exposing the coating to the hard asperities of the counterface, resulting in abrasive wear by ploughing and ultimately leading to the coating failure. It has been reported that the addition of nanofillers in the right quantity to the UHMWPE matrix helps in improving the adhesion of the transfer film to the counterface body, which in turn helps in improving the wear life of the coating.

## 4. Conclusions and Recommendations

In view of the extensive literature review presented, it may be concluded that UHMWPE polymers, because of their excellent properties such as high wear durability/resistance, low coefficient of friction, high impact strength, biocompatibility can be an excellent candidate in the form of protective coatings for metallic and polymeric substrates in demanding tribological applications. It also shows good adhesion to different substrate, which further helps in enhancing its tribological performance. As highlighted in the review, easy, adaptable and cost-effective coating techniques such as dip-coating, electrostatic spray technique and flame spraying technique have been developed for depositing the UHMWPE coatings on various substrates. With the adaptation of various approaches such as, depositing an overcoat of PFPE, introducing a hard intermediate layer, incorporation of different nanofillers either individually or in combination, researchers were able to significantly enhance the tribological performance of UHMWPE coatings to make it suitable for different tribological applications. Even though most of the studies were conducted in exploring the feasibility of using UHMWPE coatings in three major applications such as, MEMS, bearings and bio-medical the spectrum of applications remains wide open because of the excellent properties of these coatings.

However, it is to be noted that, despite of the numerous developments made in improving the tribological performance of UHMWPE coatings, a few challenges remain:

- *MEMS applications*: One of the major challenges for the suitability of UHMWPE coatings for MEMS applications is how to further reduce the thickness of the coatings without compromising their tribological performance. Hence, work could be focused on exploring various methods such as different pre-treatments, coating methodologies, post-heat treatments etc. to reduce the thickness of the UHMWPE coatings to make them more suited for MEMS applications.
- *Bearing applications*: Some of the major challenges for the suitability of the UHMWPE coatings to demanding tribological applications such as mechanical bearings is to; further increase their load bearing capacity and improve their thermal stability. Hence, different individual nanofillers or combinations can be explored further to overcome the above challenges and improve the overall suitability of UHWPE coatings to such demanding applications.
- *Biomedical applications*: One of the major concerns of using UHMWPE in the bulk form or in the form of a coating for biomedical applications is the generation of wear debris particles, which lead to other complications in the human body. Hence, different, fillers, surface treatments etc. can be explored to overcome the above challenge.

## Figures and Tables

**Table 1:** Structural, mechanical and thermal properties of UHMWPE [25].

Property	Value
**Structure**	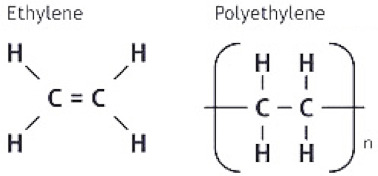
**Tensile strength**	38.6 to 43.8 MPa
**Elastic Modulus**	0.69 GPa
**Coefficient of Thermal Expansion**	234 to 360 10^−6^ °C
**Melting Temperature Range**	138–142 °C
**Working Temperature Range**	−169 °C to 90 °C
**Glass Transition Temperature**	−110 °C
**Poisson Ratio**	0.46

Data from [25].

**Table 2:** Summary of the results from Satyanayana et al. [52,53].

Sample	Thickness (µm)	Water Contact Angle (°)	Coefficient of Friction	Wear Life (cycles)
Bare Si	-	28	0.6	<100
Si/UHMWPE	28	127	0.09	~12,000
Si/UHMWPE/PFPE	28	134	0.08	>100,000
Si/GPTMS	-	52	0.46	<100
Si/GPTMS/Polar UHMWPE–With post heat treatment	1.4	109	0.1	>100,000
Si/GPTMS/Polar UHMWPE–No post heat treatment	-	-	-	6000
Si/Polar UHMWPE	1.4	-	-	3500
Si/GPTMS/UHMWPE	-	-	-	13,000

Data from [52,53].

**Table 3:** Summary of results for different samples with the thickness of UHMWPE fixed at 28 µm. Wear tests conducted a normal load of 40 mN and a sliding speed of 0.052 m/s [54].

Sample	WCA (°)	COF	Wear Life (Cycles)
Bare Si	21	0.65	5
Si/UHMWPE	93	0.18	20,000
Si/UHMWPE/PFPE	95	0.06	100,000
Si/DLC	81	0.25	400
Si/DLC/UHMWPE	91	0.13	100,000
Si/DLC/UHMWPE/PFPE	102	0.07	300,000

Data from [54].

**Table 4:** Thicknesses of the coatings obtained by using different concentrations (wt.%) of UHMWPE polymer powder in decalin. Data From [52,53,54,55,56,57].

Concentration of UHMWPE Powder (wt.%)	Thickness of the Coating (µm)
0.5	3.4
1	6.2
3	12.3
5	28

**Table 5:** Various measured parameters: WCA, RMS, Carbon content, Oxygen content–Measured before and after the pre-treatments; Wear life–Ball on disk wear tests conducted on UHMWPE coatings at a normal load of 1 N and a sliding speed of 0.042 m/s. Data From [58].

Sample Type	WCA (°)	RMS (nm)	Carbon (%)	Oxygen (%)	Wear life (Cycles)
Si-Untreated	38.4	0.53	17.7	37.6	500
Si–Piranha Treated	21.3	0.25	19.7	34.3	2000
Si–Air plasma treated	4.3	0.29	16.9	42.9	50,000

**Table 6:** Properties of UHMWPE nanocomposite coating reinforced with 0.1 wt.% of CNTs. Data From [61].

Property	Value
Water contact angle (°)	139 ± 3
Thickness (µm)	55 ± 4
Hardness (MPa)	97 ± 9
Elastic modulus (GPa)	3.76 ± 0.55
Thermal conductivity (W/mK)	0.64 ± 0.03

**Table 7:** Summary of the literature of UHMWPE coatings for MEMS applications.

Ref	Pre-Treatment	Coating Type	Operating Conditions		Counter face (Dia/mm, Ra/nm)	Wear Life (Cycles)	COF	UHMWPE Coating Thickness (µm)	Remarks
			Load (N)	Speed (m/s)					
[52]	Piranha	Si/UHMWPE/PFPE	0.07	0.042	Si_3_N_4_ (4, 20)	>100,000	0.06	28	UHMWPE coatings were dip-coated on Si substrates. An overcoat of PFPE was deposited on the UHMWPE coatings to form a dual layer coating of Si/UHMWPE/PFPE Ball on disk wear tests were conducted They found a significant increase in the wear life as the dual layer coating did not fail even until 100,000 cycles, as compared to Si/UHMWPE (~12,000 cycles, COF = 0.09) and bare Si (~100 cycles, COF = 0.6) The wear life was defined as the number of cycles after which the coefficient of friction exceeded a value of 0.3 or a visible wear scar appeared on the substrate, whichever happened earlier. They attributed the improvement to the high wear resistance of UHMWPE and the lubricious nature of PFPE
[53]	Piranha	Si/GPTMS/Polar UHMWPE	0.3	0.042	Si_3_N_4_ (4, 20)	~6000	0.6	1.4	UHMWPE coatings were dip-coated on Si substrates with an intermediate self-assembles monolayer of GPTMS. Ball on disk wear tests were conducted They found the Si/GPTMS / Polar UHMWPE / Post-heat treated (110 °C) coating extremely wear resistant, as it did not fail even until 100,000 cycles, as compared to Si/GPTMS/Polar UHMWPE (~6000 cycles, COF = 0.6) The wear life was defined as the number of cycles after which the coefficient of friction exceeded a value of 0.3 or a visible wear scar appeared on the substrate, whichever happened earlier. They attributed the improvement to the chemisorption of polar UHMWPE molecules to substrate because of the intermediate GPTMS layer.
		Si/GPTMS/Polar UHMWPE/Post-heat treated (110 °C)	0.3	0.042	Si_3_N_4_ (4, 20)	>100,000	0.1	1.4	
[54]	Piranha	Si/DLC	0.04	0.052	Si_3_N_4_ (4, 20)	400	0.25	-	UHMWPE coatings were dip-coated on Si substrates with an intermediate of DLC of 50 nm thickness. Ball on disk wear tests were conducted They found the Si/DLC/UHMWPE / PFPE to be extremely wear resistant, as it did not fail even until 300,000 cycles. The wear life was defined as the number of cycles after which the coefficient of friction exceeded a value of 0.3 or a visible wear scar appeared on the substrate, whichever happened earlier. They attributed the improvement to the load bearing capacity provided by the hard intermediate layer of DLC, the self-lubricating property of UHMWPE and the thermal stability of PFPE.
		Si/UHMWPE	0.04	0.052	Si_3_N_4_ (4, 20)	20,000	0.18	28	
		Si/DLC/UHMWPE	0.04	0.052	Si_3_N_4_ (4, 20)	100,000	0.13	28	
		Si/DLC/UHMWPE/PFPE	0.04	0.052	Si_3_N_4_ (4, 20)	>300,000	0.06	28	
[55]	Piranha	Si/CrN (hardness = 13.5 GPa)/UHMWPE	0.04	0.052	Si_3_N_4_ (4, 20)	2000	0.25	5	UHMWPE coatings were dip-coated on Si substrates with an intermediate of increasing hardness of CrN, DLC15, TiN, DLC57, DLC70 of 50 nm thickness. Ball on disk wear tests were conducted They found the wear resistance of UHMWPE coatings to increase with the increase in the hardness of the intermediate layer. The wear life was defined as the number of cycles after which the coefficient of friction exceeded a value of 0.3 or a visible wear scar appeared on the substrate, whichever happened earlier. The highest wear resistance was showed by Si/DLC (hardness = 70 GPa)/UHMWPE coating. When an overcoat of PFPE was deposited, the wear resistance increased further even at a higher load of 70 mN. They attributed the improvement to the load bearing capacity provided by the hard intermediate layer of DLC, the self-lubricating property of UHMWPE and the thermal stability of PFPE.
		Si/DLC (hardness = 15 GPa)/UHMWPE	0.04	0.052	Si_3_N_4_ (4, 20)	50,000	0.14	5	
		Si/TiN (hardness = 24 GPa)/UHMWPE	0.04	0.052	Si_3_N_4_ (4, 20)	100,000	0.13	5	
		Si/DLC (hardness = 57 GPa)/UHMWPE	0.04	0.052	Si_3_N_4_ (4, 20)	100,000	0.18	5	
		Si/DLC (hardness = 70 GPa)/UHMWPE	0.04	0.052	Si_3_N_4_ (4, 20)	100,000	0.05	5	
[57]	-	Si (WCA = 21°)/UHMWPE	0.04	0.1	Si_3_N_4_ (4, 20)	1000	0.17	6	UHMWPE coatings were dip-coated on Si substrates with different surface energies and wettability. Ball on disk wear tests were conducted They found the wear resistance of UHMWPE coatings deposited on the hydrogenated Si substrates with a WCA = 71° to be the highest (250,000 cycles) However, as the wettability increase further, the wear life decreased significantly to 6500 cycles. The wear life was defined as the number of cycles after which the coefficient of friction exceeded a value of 0.3 or a visible wear scar appeared on the substrate, whichever happened earlier. The finally concluded that by simply introducingan interface with optimum wettability, the tribologicalperformance of the UHMWPE coating can be increased by several orders of magnitude
		Si (heated) (WCA = 46°)/UHMWPE	0.04	0.1	Si_3_N_4_ (4, 20)	60,000	0.17	6	
		Si / APTMS (WCA = 52°)/UHMWPE	0.04	0.1	Si_3_N_4_ (4, 20)	100,000	0.17	6	
		Si-H (WCA = 71°)/UHMWPE	0.04	0.1	Si_3_N_4_ (4, 20)	250,000	0.17	6	
		Si/OTS (WCA = 104°)/UHMWPE	0.04	0.1	Si_3_N_4_ (4, 20)	6500	0.17	6	
[58]	Piranha and Air-plasma treatment	Si (Untreated)/UHMWPE	1	0.042	Si_3_N_4_ (4, 20)	500	-	16.3	UHMWPE coatings were dip-coated on Si substrates with different pre-treatments, such as Piranha and air-plasma treatment. Ball on disk wear tests were conducted. The UHMWPE coating deposited on the air-plasma treated Si substrate showed an increased wear life of ~50,000 cycles as compared to the coating deposited on piranha treated Si substrate (~2000 cycles). The wear life was defined as the number of cycles after which the coefficient of friction exceeded a value of 0.3 or a visible wear scar appeared on the substrate, whichever happened earlier. They attributed this increase to the carbon cleaning effect and the oxidizing effect of air-plasma treatment leading to an increase in the adhesion between the coating and the substrates.
		Si (Piranha)/UHMWPE	1	0.042	Si_3_N_4_ (4, 20)	2000	-	15.2	
		Si (Air-Plasma)/UHMWPE	1	0.042	Si_3_N_4_ (4, 20)	50,000	0.1	16.6	

**Table 8:** Summary of the literature of UHMWPE coatings for mechanical bearing applications.

Ref	Substrate/ Pre-Treatment	Fillers (wt.%)/ Dispersion Technique	Operating Conditions		Type of Test/Counter Face (Dia mm)	Wear Life (Cycles)	COF	Coating Thickness (µm)/ Deposition Technique	Remarks
			Load (N)	Speed (m/s)					
[60]	DF3 Tool Steel/Air-Plasma Treated	-	0.3	0.02	Ball on disk/Ball: Si_3_N_4_ (4)	>100,000	0.14	16.3/Dip-Coating	Pristine UHMWPE coatings were dip-coated on air-plasma treated DF3 Tool steel substrates. The optimum thickness of the UHMWPE coating was found to be 16.3 µm as it did not fail even until 100,000 cycles. An overcoat of PFPE was deposited on the UHMWPE coatings to form a dual layer coating of DF3 Tool Steel/UHMWPE/PFPE They found a significant increase in the wear life as the dual layer coating did not fail even until 200,000 cycles, at a load of 4 N and at a speed of 0.1 m/s The wear life was defined as the number of cycles after which the coefficient of friction exceeded a value of 0.3 or a visible wear scar appeared on the substrate, whichever happened earlier. They attributed the improvement to the high wear resistance of UHMWPE and the lubricious nature of PFPE
[61]	DF3 Tool Steel/Air-Plasma Treated	Carbon Nanotubes (CNTs: 0.05, 0.1, 0.2)/Sonication and magnetic stirring	4	0.26	Ball on disk/Ball: Si_3_N_4_ (4)	>10 million	0.16	55 ± 4/Dip-Coating	UHMWPE nanocomposite coatings with different concentrations of CNTs were dip-coated on air-plasma treated DF3 Tool Steel substrates. A significant improvement in hardness (66%) and elastic modulus (58%) was observed for 0.2 wt.% reinforced UHMWPE nanocomposite coating. The wear life increased by two orders of magnitude as compared to pristine UHMWPE coating. The wear life was defined as the number of cycles after which the coefficient of friction exceeded a value of 0.3 or a visible wear scar appeared on the substrate, whichever happened earlier. They attributed the improvement to the inherent excellent mechanical properties of CNTs.
[62]	AISI 52100 Bearing Steel/Air-Plasma Treated	Carbon Nanotubes (CNTs: 0.1)/Sonication and magnetic stirring	4	0.52	Ball on disk / Ball: - Si_3_N_4_ (4) - Brass (4) - Stainless Steel (4)	>1 million	0.11	55 ± 4/Dip-Coating	The tribological performance of the nanocomposite coating was independent of the counterface material The wear performance of the developed nanocomposite coating was excellently resistant to UV radiation (0.71 W/m^2^ nm for 300 h) The wear life was defined as the number of cycles after which the coefficient of friction exceeded a value of 0.3 or a visible wear scar appeared on the substrate, whichever happened earlier. They attributed the improvement to the inherent excellent mechanical properties of CNTs. An overcoat of PFPE further increased the tribological performance of the nanocomposite coating
[63]	AISI 52100 Bearing Steel/Air-Plasma Treated	Carbon Nanotubes (CNTs: 0.1)/Sonication and magnetic stirring	60	0.11	Plate on cylinder/Plate: AISI 52100 bearing steel	150,000	0.14	55 ± 4/Dip-Coating	UHMWPE nanocomposite coatings were dip-coated on air-plasma treated AISI bearing Steel cylindrical shafts. The nanocomposite coating did not fail even until 150,000 cycles under dry conditions at room temperature An overcoat of PFPE reduced the COF from 0.14 to 0.09 The nanocomposite coating was able to sustain higher temperatures until 120 °C under dry conditions. The nanocomposite coating with a PFPE overcoat was able to sustain a temperature of 105 °C under base oil lubricated conditions. The wear life was defined as the number of cycles after which the coefficient of friction exceeded a value of 0.3 or a visible wear scar appeared on the substrate, whichever happened earlier. They attributed the improved thermal performance of the nanocomposite coatings to the excellent thermal properties of CNTs and the thermal stability of PFPE.
[64]	Aluminum/Air-Plasma Treated	Carbon Nanotubes (CNTs: 0.1)/Sonication and magnetic stirring	45	0.57	Plate on cylinder/Plate: Aluminum (Dry conditions)	> 2 million	0.17	55 ± 4/Dip-Coating	UHMWPE nanocomposite coatings were dip-coated on air-plasma treated aluminum cylindrical shafts. The nanocomposite coating did not fail even until 2 million cycles under dry conditions at room temperature The nanocomposite coating performed excellently well under both dry and base oil lubricated conditions. The nanocomposite coating’s mechanical properties did not change even after a test of 100 h duration. The wear life was defined as the number of cycles after which the coefficient of friction exceeded a value of 0.3 or a visible wear scar appeared on the substrate, whichever happened earlier. They attributed the improved tribological performance of the nanocomposite coatings to the excellent properties of CNTs and their uniform dispersion.
			60	0.11	Plate on cylinder/Plate: Aluminum (Base oil Lubricated conditions)	> 3 million	0.11	55 ± 4/Dip-Coating	
[65]	304L Stainless steel/Grid Blasted/pre-heated to 110–130 °C	Graphene Nanosheets (GNs: 0.15, 0.3, 1)	35	0.01	Ball on disk/Ball: 304L Stainless steel (9.5)	-	0.18	Flame spraying	UHMWPE nanocomposite coatings reinforced with GNs were flame sprayed on grid blasted and pre-heated stainless steel samples The COF reduced significantly from 0.24 (pristine UHMWPE) to 0.18 (1 wt.% GN nanocomposite coating) The wear resistance significantly increased with the 1 wt.% GN nanocomposite coating showing the lowest wear rate of 0.0011 mm^3^/N.m The corrosion resistance of the 1 wt.% GN nanocomposite coating showed a significant improvement.
[66]	PEEK/Air-Plasma treated	-	7	0.1	Ball on disk/Ball: 440C Stainless steel (6.3)	250,000	0.09	27 ± 2/Dip-Coating	UHMWPE and UHMWPE/CNTs nanocomposite coatings were dip-coated on air-plasma treated PEEK substrates. The UHMWPE coating was able to reduce the COF of PEEK from o.3 to 0.09 and did not fail even until 250,000 cycles. The nanocomposite coating with 0.2 wt.% CNTs performed excellently well with increased load bearing capacity of 9 N and higher sliding speeds of 0.2 m/s The wear life was defined as the number of cycles after which the coefficient of friction exceeded a value of 0.3 or a visible wear scar appeared on the substrate, whichever happened earlier. They attributed the improved tribological performance of the nanocomposite coatings to the excellent properties of CNTs and their uniform dispersion.
[67]	PEEK/Air-Plasma treated	Carbon Nanotubes (CNTs: 0.1 and 0.2)/Sonication and magnetic stirring	9	0.2	Ball on disk/Ball: 440C Stainless steel (6.3)	>25,000	0.09	7.5 ± 2/Dip-Coating	
[68]	Aluminum/Air-Plasma treated/pre-heat treated at 180 °C (5 min)	Nanoclay (C15A: 0.5, 1.5 and 3)/Sonication and magnetic stirring	9	0.1	Ball on disk/Ball: 440 C Stainless steel (6.3)	>100,000	0.16	125 ± 4/Electrostatic spraying	UHMWPE/C15A nanocomposite coatings were electrostatically spray coated on air-plasma and pre-heat treated Aluminum substrates. UHMWPE/1.5 wt.% C15A shoed the best tribological performance at it did not fail even until 100,000 cycles at a load of 9 N and a speed of 0.1 m/s The wear life was defined as the number of cycles after which the coefficient of friction exceeded a value of 0.3 or a visible wear scar appeared on the substrate, whichever happened earlier. They attributed the improved tribological performance of the nanocomposite coatings to the excellent properties of nanoclay and their uniform dispersion leading to an exfoliated morphology.
[69]	Aluminum/Air-Plasma treated	Graphene Nanoplatelets (GNPs: 0.25, 1 and 2)/Sonication and magnetic stirring	15	0.1	Pin on disk/Pin: Hardened tool steel (3)	-	0.3	96 ± 4.7/Electrostatic spraying	The UHMWPE nanocomposite coating reinforced with 1 wt.% GNPs showed the lowest wear rate of 18.2 µm/km corresponding to a 51% reduction as compared to the pristine UHMWPE coating. The overall PV limit of the UHMWPE/1 wt.% GNPs nanocomposite coating was found to be 4 MPa m/s which is much higher than the PV limits of some commercially available materials.
[70]	Aluminum/Air-Plasma treated	Graphene Nanoplatelets (GNPs: 1)/Sonication and magnetic stirring	Contact Pressures: 0.1, 0.5, 0.8, 1 MPa	1, 1.5, 2	Ring on disk/Disk: AISI 4140 steel (125) (dry and lubricated conditions)	-	-	94 ± 6/Electrostatic spraying	The COF of UHMWPE nanocomposite coating reinforced with 1 wt.% GNPs reduced from 0.42 to 0.33 with an increase in sliding speed, under dry conditions. UHMWPE/1 wt.% GNPs coating sustained a higher maximum contact pressure of 0.8 MPa as compared to pristine UHMWPE coating that could only sustain 0.3 MPa, in dry conditions. The UHMWPE/1 wt.% GNPs coating sustained a maximum contact pressure of 2.7 MPa as compared to the pristine UHMWPE coating which sustained only 0.5 MPa, under base oil lubricated conditions.
[71]	AA2028 Al alloy/Air-Plasma treated	Graphene Nanoplatelets (GNPs: 0.5, 1 and 2)/Sonication and magnetic stirring	35 N	0.5	Pin on disk / Pin: Silver Steel BS-1407 (3)	-	-	99.34 / Electrostatic spraying	The UHMWPE coatings reinforced with 1 wt. % GNPs performed best with a maximum reduction in friction and wear by 29 and 36 % respectively. The coating withstood a maximum sliding speed of 2.6 m/s without failing which corresponds to a PV value 13 MPa.m/s. The UHMWPE coatings reinforced with 2 wt. % GNPs showed enhanced corrosion performance in 3.5% NaCl solution.
[72]	Steel/Air-Plasma treated/pre-heat treated at 180 °C (5 min)	Alumina (Al_2_O_3_: 0.5, 3, 5 and 10)/Sonication and magnetic stirring	12	0.1	Ball on disk/Ball: 440C Stainless steel (6.3)	>250,000	0.13	60 ± 3/Electrostatic spraying	Nanocomposite coatings with 3 and 5 wt.% exhibited the highest wear resistance as they did not fail even until 250,000 cycles at a load of 12 N. This is attributed to the superior mechanical properties of alumina, and its uniform dis.persion in the polymer matrix. A combination of adhesive and abrasive wear was found to be the major wear mechanisms.
[73]	Copper (Cu)	Nickel	2	0.1	Ball on disk/Ball: 304L Stainless steel (4)	-		8 ± 1/Eletrophoric/Electro plating deposition	Coatings with the higher UHMWPE particle content showed better antifriction and anti-wear performance. In comparison to the co-deposited coatings, tribological performance of the coating produced via the two-step method was much better. The wear of the counterface ball reduced.
[74]	Low alloyed Steel/Air-Plasma treated /pre-heat treated at 180 °C (5 min)	Tungsten arbide (WC: 1, 3, 6 and 9)/Sonication and magnetic stirring	-	-	-	-	-	85 ± 5/Electrostatic spraying	The scratch and corrosion resistance of the UHMWPE nanocomposite coatings reinforced with 6 wt.% of WC particles was the best.
[75]	Aluminum / Air-Plasma treated/pre-heat treated at 180 °C (5 min)	HYBRID–Nanoclay (C15A: 1.5) + Carbon Nanotubes (CNTs: 0.5, 1.5 and 3)/Sonication and magnetic stirring	12	0.1	Ball on disk/Ball: 304L Stainless steel (4)–Dry conditions	>100,000	0.16	180 ± 3/Electrostatic spraying	The hybrid nanocomposite coating (UHMWPE/1.5 wt.% C15A/1.5 wt.% CNTs) showed an enhanced load bearing capacity of 12 N as compared to the nanocomposite coating (UHMWPE/1.5 wt.% C15A–9 N) and the pristine UHMWPE coating (7 N). It showed a wear life of 100,000 cycles at a load of 12 N and a speed of 0.1 m/s. The increase in the wear life is attributed to efficient dispersion of nanoclay and CNTs, which provide the bridging and anchoring of the polymer chains in the matrix resisting their pull out.
[76]	Aluminum/Air-Plasma treated/pre-heat treated at 180 °C (5 min)	HYBRID–Nanoclay (C15A: 1.5) + Carbon Nanotubes (CNTs: 0.5, 1.5 and 3)/Sonication and magnetic stirring	12	0.1	Ball on disk/Ball: 304L Stainless steel (4)–Water lubricated conditions	300,000	0.12	180 ± 3/Electrostatic spraying	The hybrid nanocomposite coating (UHMWPE/1.5 wt.% C15A/1.5 wt.% CNTs) did not fail under water even until 300,000 cycles at a load of 12 N and a speed of 0.1 m/s. The improved performance under water is attributed to the presence of nanoclay because of its platelet structure presents a torturous path for the diffusion of water though the polymer matrix. The tribological performance of the hybrid coating improved under water with abrasives as well.

**Table 9:** Summary of the literature of UHMWPE coatings for bio-medical applications.

Ref	Substrate/ Pre-Treatment	Fillers (wt.%)/Dispersion Technique	Operating Conditions		Type of Test/Counter Face (Dia mm)	Wear Life (Cycles)	COF	Coating Thickness (µm)/Deposition Technique	Remarks
			Load (N)	Speed (m/s)					
[79]	Titanium alloy (Ti6Al4V) /Air-Plasma Treated	-	4	0.083	Ball on disk/Ball: Si_3_N_4_ (4)	>175,000	0.10	19.6 ± 2.0/Dip-Coating (withdrawal speed = 1.9 mm/s, soaking time = 35 s)	Pristine UHMWPE coatings were dip-coated on air-plasma treated titanium alloy substrates. The UHMWPE coatings showed an excellent wear life of 175,000 cycles at a load of 4 N and a speed of 0.08 m/s. An overcoat of PFPE was deposited on the UHMWPE coatings to form a dual layer coating of Ti/UHMWPE/PFPE They found a significant increase in the wear life as the dual layer showed a wear life of 60,000 cycles, at a load of 4 N and as speed of 0.2 m/s as compared to the pristine UHMWPE coating which failed at 28,000 cycles. The wear life was defined as the number of cycles after which the coefficient of friction exceeded a value of 0.3 or a visible wear scar appeared on the substrate, whichever happened earlier. The UHMWPE coating exhibited a cytotoxicity level of grade 0 (reactivity: none) according to test results and met the requirements of the ISO elution method-1XMEM extract since grade was less than the grade 2 (mild reactivity). The combination of hydrophobicity, wear durability and nontoxicity makes this coating a potential candidate for biomedical applications.
[80]	Titanium alloy (Ti6Al4V)/Air-Plasma Treated	HYBRID–Carbon Nanotubes (CNTs: 0.5, 1.5 and 3) + Hydroxyapatite (HA: 0.5, 1.5, 3 and 5)/Sonication and magnetic stirring	7, 9, 12, 15	0.1	Ball on disk/Ball: 440C Stainless steel (6.3)	250,000	0.15	181 ± 4/Electrostatic spraying technique	The hybrid nanocomposite coating (UHMWPE/1.5 wt.% CNTs/3 wt.% HA) showed an enhanced load bearing capacity of 12 N and at a sliding speed of 0.1 m/s. The increase in the wear life is attributed to efficient dispersion of CNTs and HA which provide the bridging and anchoring of the polymer chains in the matrix resisting their pull out.

## References

[1] Vižintin J., Kalin M., Dohda K. (2004). Tribology of Mechanical Systems: A Guide to Present and Future Technologies.

[2] Gold J., Loos W.P. (2002). Wear resistance of PVD coatings in roller bearings. Wear.

[3] Erdemir F.A., Nichols X.Z., Pan R.W., Wilbur P. (1994). Friction and wear performance of ion-beam-deposited diamond-like carbon films on steel substrates. Diam. Relat. Mater..

[4] Grill A. (1997). Tribology of diamondlike carbon and related materials: An updated review. Surf. Coatings Technol..

[5] Gåhlin R., Larsson M., Hedenqvist P. (2001). ME-C:H coatings in motor vehicles. Wear.

[6] Siu J.H.W., Li L.K.Y. (2000). An investigation of the effect of surface roughness and coating thickness on the friction and wear behaviour of a commercial MoS 2–metal coating on AISI 400C steel. Wear.

[7] Jiménez A.E., Bermúdez M.D., Carrión F.J., Nicolás M.G. (2006). Room temperature ionic liquids as lubricant additives in steel-aluminium contacts: Influence of sliding velocity, normal load and temperature. Wear.

[8] Glovnea R.P., Olver A.V., Spikes H.A. (2005). Lubrication of Rough Surfaces by a Boundary Film-Forming Viscosity Modifier Additive. J. Tribol..

[9] Monaghan D.P., Teer D.G., Logan P.A., Efeoglu I., Arnell R.D. (1993). Deposition of wear resistant coatings based on diamond like carbon by unbalanced magnetron sputtering. Surf. Coat. Technol..

[10] Unal H., Mimaroglu A., Kadioglu U., Ekiz H. (2004). Sliding friction and wear behaviour of polytetrafluoroethylene and its composites under dry conditions. Mater. Des..

[11] Lee J.Y., Lim D.S. (2004). Tribological behavior of PTFE film withnanodiamond. Surf. Coat. Technol..

[12] McCook N.L., Burris D.L., Bourne G.R., Steffenes J., Hanrahan J.R., Sawyer W.G. (2005). Wear resistant solid lubricant coating made from PTFE and epoxy. Tribol. Lett..

[13] Lu Z.P., Friedrich K. (1995). On sliding friction and wear of PEEK. Wear.

[14] Hanchi J., Eiss N.S. (1997). Dry sliding friction and wear of short carbon-fiber-reinforced polyetheretherketone (PEEK) at elevated temperatures. Wear.

[15] Corni I., Neumann N., Eifler D., Boccaccini A.R. (2008). Polyetheretherketone (PEEK) Coatings on Stainless Steel by Electrophoretic Deposition. Adv. Eng. Mater..

[16] Tailor S., Vashistha N., Modi A., Modi S.C. (2020). One-step fabrication of thermal sprayed polymer coating on metals. Mater. Res. Express.

[17] Satheesan B., Mohammed A.S. (2021). Tribological characterization of epoxy hybrid nanocomposite coatings reinforced with graphene oxide and titania. Wear.

[18] Bobby S., Samad M.A. (2016). Enhancement of tribological performance of epoxy bulk composites and composite coatings using micro/nano fillers: A review. Polym. Adv. Technol..

[19] Satheesan B., Mohammed A.S. (2018). Evaluation of parameters affecting adhesive strength of high build epoxy coatings on textured and plasma treated metallic substrates. J. Adhes. Sci. Technol..

[20] Kumar V., Sinha S.K., Agarwal A.K. (2015). Tribological studies of epoxy and its composite coatings on steel in dry and lubricated sliding. Tribol. Mater. Surface Interfac..

[21] Li Y., Ma Y., Xie B., Cao S., Wu Z. (2007). Dry friction and wear behavior of flame-sprayed polyamide1010/n-SiO2 composite coatings. Wear.

[22] Bello J.O., Wood R.J.K. (2005). Micro-abrasion of filled and unfilled polyamide 11 coatings. Wear.

[23] Friedrich K. (2018). Polymer composites for tribological applications. Adv. Ind. Eng. Polym. Res..

[24] Lampman S. (2003). Characterization and Failure Analysis of Plastics.

[25] Kurtz S.M. (2004). Uhmwpe Handbook.

[26] Lewis G. (2001). Properties of crosslinked ultra-high-molecular-weight polyethylene. Biomaterials.

[27] Park K., Mishra S., Lewis G., Losby J., Fan Z., Park J.B. (2004). Quasi-static and dynamic nanoindentation studies on highly crosslinked ultra-high-molecular-weight polyethylene. Biomaterials.

[28] Karuppiah K.K.S., Bruck A.L., Sundararajan S., Wang J., Lin Z., Xu Z.H., Li X. (2008). Friction and wear behavior of ultra-high molecular weight polyethylene as a function of polymer crystallinity. Acta Biomater..

[29] Fouad H., Mourad A.H.I., Barton D.C. (2005). Effect of pre-heat treatment on the static and dynamic thermo-mechanical properties of ultra-high molecular weight polyethylene. Polym. Test..

[30] Di Y., Gang X., Chunhua C. (2014). The effect of gamma irradiation on the tribological properties of UHMWPE composite filled with HDPE. J. Thermoplast. Compos. Mater..

[31] Zhang J. (2014). Surface modification of ultra-high-molecular-weight polyethylene by argon plasma. J. Thermoplast. Compos. Mater..

[32] Liu H., Pei Y., Xie D., Deng X., Leng Y.X., Jin Y., Huang N. (2010). Surface modification of ultra-high molecular weight polyethylene (UHMWPE) by argon plasma. Appl. Surf. Sci..

[33] Kovalchenko A., Ajayi O., Erdemir A., Fenske G., Etsion I. (2005). The effect of laser surface texturing on transitions in lubrication regimes during unidirectional sliding contact. Tribol. Int..

[34] Etsion I. (2004). Improving Tribological Performance of Mechanical Components by Laser Surface Texturing. Tribol. Lett..

[35] Mohammed A.S., Ali A.B., Nesar M. (2016). Evaluation of Tribological properties of Organo-clay reinforced UHMWPE Nanocomposites. J. Tribol..

[36] Mohammed A.S., Ali A.B., Merah N. (2016). Investigating the Effect of Water Uptake on the Tribological Properties of Organoclay Reinforced UHMWPE Nanocomposites. Tribol. Lett..

[37] Ali A.B., Samad M.A., Merah N. (2016). Tribological investigations of UHMWPE nanocomposites reinforced with three different organo-modified clays. Polym. Compos..

[38] Ali A.B., Mohammed A.S., Nesar M. (2017). UHMWPE Hybrid Nanocomposites for Improved Tribological Performance under Dry and Water Lubricated Sliding Conditions. Tribol. Lett..

[39] Pruitt L.A. (2005). Deformation, yielding, fracture and fatigue behavior of conventional and highly cross-linked ultra high molecular weight polyethylene. Biomaterials.

[40] Puértolas J.A., Kurtz S.M. (2014). Evaluation of carbon nanotubes and graphene as reinforcements for UHMWPE-based composites in arthroplastic applications: A review. J. Mech. Behav. Biomed. Mater..

[41] Baena J., Wu J., Peng Z. (2015). Wear Performance of UHMWPE and Reinforced UHMWPE Composites in Arthroplasty Applications: A Review. Lubricants.

[42] Spearing S.M. (2000). Materials issues in microelectromechanical systems. Acta Mater..

[43] Tsukruk V.V. (2001). Molecular lubricants and Glues for Micro- and Nanodevices. Adv. Mater..

[44] Ahn H.S., Julthongpiput D., Kim D.I., Tsukruk V.V. (2003). Dramatic enhancement of wear stability in oil-enriched polymer gel nanolayers. Wear.

[45] Satyanarayana N., Sinha S.K., Shen L. (2007). Effect of molecular structure on friction and wear of polymer thin films deposited on Si surface. Tribol. Lett..

[46] Satyanarayana N., Lau K.H., Sinha S.K. (2008). Nanolubrication of poly (methyl methacrylate) films on Si for microelectromechanical systems applications. Appl. Phys. Lett..

[47] Nakano M., Ishida T., Numata T., Ando Y., Sasaki S. (2005). Tribological behavior of self assembled double layer measured by a pin-on-plate method. Appl. Surf. Sci..

[48] Sidornko A., Ahn H., Kim D., Yang H., Tsukruk V.V. (2002). Wear stability of polymer nanocomposite coatings with trilayer architecture. Wear.

[49] Julthongpiput D., Ahn H.S., Kim D.I., Tsukruk V.V. (2002). Tribological behavior of grafted polymer gel nanocoatings. Tribol. Lett..

[50] Kim D.I., Zhavnerko G.K., Ahn H.S., Choi D.H. (2004). Tribological properties of Langmuir Blodgett films on silicon surface in microscale sliding contact. Tribol. Lett..

[51] Cha K.H., Kim D.E. (2001). Investigation of the tribological behavior of octadecyltrichlorosilane deposited on silicon. Wear.

[52] Satyanarayana N., Sinha S.K., Ong B.H. (2006). Tribology of a novel UHMWPE/PFPE dual-film coated onto Si surface. Sens. Actuators A.

[53] Satyanarayana N., Sinha S.K., Lim S. (2009). Highly wear resistant chemisorbed polar ultra-high-molecular-weight polyethylene thin film on Si surface for micro-system applications. J. Mater. Res..

[54] Minn M., Sinha S.K. (2008). DLC and UHMWPE as hard/soft composite film on Si for improved tribological performance. Surf. Coat. Technol..

[55] Minn M., Sinha S.K. (2010). Tribology of ultra high molecular weight polyethylene film on Si substrate with chromium nitride, titanium nitride and diamond like carbon as intermediate layers. Thin Solid Film..

[56] Minn M., Sinha S.K. (2009). Molecular orientation, crystallinity, and topographical changes in sliding and their frictional effects for UHMWPE film. Tribol. Lett..

[57] Minn M., Jonathan L.Y., Sinha S.K. (2008). Effects of interfacial energy modifications on the tribology of UHMWPE coated Si. J. Phys. D Appl. Phys..

[58] Samad M.A., Satyanarayana N., Sinha S.K. (2010). Effect of Air–Plasma Pre-treatment of Si Substrate on Adhesion Strength and Tribological Properties of a UHMWPE Film. J. Adhes. Sci. Technol..

[59] Possart W. (2005). Adhesion: Current Research and Applications.

[60] Samad M.A., Satyanarayana N., Sinha S.K. (2010). Tribology of UHMWPE film on air-plasma treated tool steel and the effect of PFPE overcoat. Surf. Coat. Technol..

[61] Samad M.A., Sinha S.K. (2011). Mechanical, thermal and tribological characterization of a UHMWPE film reinforced with carbon nanotubes coated on steel. Tribol. Int..

[62] Samad M.A., Sinha S.K. (2011). Effects of counterface material and UV radiation on the tribological performance of a UHMWPE/CNT nanocomposite coating on steel substrates. Wear.

[63] Samad M.A., Sinha S.K. (2011). Dry sliding and boundary lubrication performance of a UHMWPE/CNTs nanocomposite coating on steel substrates at elevated temperatures. Wear.

[64] Samad M.A., Sinha S.K. (2010). Nanocomposite UHMWPE–CNT Polymer Coatings for Boundary Lubrication on Aluminium Substrates. Tribol. Lett..

[65] Han J., Ding S., Zheng W., Li W., Li H. (2013). Microstructure and anti- wear and corrosion performances of novel UHMWPE/graphenenanosheet composite coatings deposited by flame spraying. Polym. Adv. Technol..

[66] Mohammed A.S., Fareed M.I. (2017). Surface Modification of Polyether Ether Ketone (PEEK) with a Thin Coating of UHMWPE for Better Tribological Properties. Tribol. Trans..

[67] Mohammed A.S., Fareed M.I. (2016). Improving the friction and wear of poly-ether-etherketone (PEEK) by using thin nano-composite coatings. Wear.

[68] Azam M.U., Samad M.A. (2018). A novel organoclay reinforced UHMWPE nanocomposite coating for tribological applications. Prog. Org. Coat..

[69] Aliyu I.K., Mohammed A.S., Qutub A.A. (2018). Tribological Performance of UHMWPE/GNPs Nanocomposite Coatings for Solid Lubrication in Bearing Applications. Tribol. Lett..

[70] Aliyu I.K., Samad M.A., Qutub A.A. (2021). Tribological characterization of a bearing coated with UHMWPE/GNPs nanocomposite coating. Surf. Eng..

[71] Aliyu I.K., A M.K., Mohammed A.S. (2021). Wear and corrosion resistance performance of UHMWPE/GNPs nanocomposite coatings on AA2028 Al alloys. Prog. Org. Coat..

[72] Mohammed A.S. (2018). UHMWPE Nanocomposite Coatings Reinforced with Alumina (Al2O3) Nanoparticles for Tribological Applications. Coatings.

[73] Guo L., Dai Q., Huang W., Wang X. (2019). Composite Ni/UHMWPE coatings and their tribological performances. Appl. Surf. Sci..

[74] Adesina A.Y., Khan M.F., Azam M.U., Samad A., Sorour A.A. (2019). Characterization and corrosion resistance of ultra-high molecular weight polyethylene composite coatings reinforced with tungsten carbide particles in hydrochloric acid medium. J. Polym. Eng..

[75] Azam M.U., Samad M.A. (2018). Tribological Evaluation of a UHMWPE Hybrid Nanocomposite Coating Reinforced with Nanoclay and Carbon Nanotubes Under Dry Conditions. J. Tribol..

[76] Azam M.U., Samad M.A. (2018). UHMWPE hybrid nanocomposite coating reinforced with nanoclay and carbon nanotubes for tribological applications under water with/without abrasives. Tribol. Int..

[77] Hussain M., Naqvi R.A., Abbas N., Khan S.M., Nawaz S., Hussain A., Zahra N., Khalid M.W. (2020). Ultra-High-Molecular-Weight-Polyethylene (UHMWPE) as a Promising Polymer Material for Biomedical Applications: A Concise Review. Polymers.

[78] Hussein M.A., Mohammed A.S., Aqeeli A.N. (2015). Wear Characteristics of Metallic Biomaterials: A Review. Materials.

[79] Panjwani B., Satyanarayana N., Sinha S.K. (2011). Tribological characterization of a biocompatible thin film of UHMWPE on Ti6Al4V and the effects of PFPE as top lubricating layer. J. Mech. Behav. Biomed. Mater..

[80] Baduruthamal Z.A., Mohammed A.S., Kumar M., A H.M., Aqeeli A.N. (2019). Tribological and Electrochemical Characterization of UHMWPE Hybrid Nanocomposite Coating for Biomedical Applications. Materials.

